# Short‐Term, Mid‐Term, and Long‐Term Outcomes of Transcatheter Aortic Valve Replacement With Balloon‐Expandable Versus Self‐Expanding Valves: A Meta‐Analysis of Randomized Controlled Trials

**DOI:** 10.1002/clc.70134

**Published:** 2025-04-19

**Authors:** Kiarash Tavakoli, Negin Sadat Hosseini Mohammadi, Pegah Bahiraie, Sahar Saeidi, Farhad Shaker, Arman Soltani Moghadam, Sara Montazeri Namin, Habib Rahban, Shubhadarshini Pawar, Masih Tajdini, Hamidreza Soleimani, Yaser Jenab, Yousif Ahmad, Fady Hany Iskander, Mohamad Alkhouli, Raj Makkar, Aakriti Gupta, Kaveh Hosseini

**Affiliations:** ^1^ Tehran Heart Center, Cardiovascular Diseases Research Institute Tehran University of Medical Sciences Tehran Iran; ^2^ School of Medicine Shahid Beheshti University of Medical Sciences Tehran Iran; ^3^ Cardiovascular Research Foundation of Southern California Beverly Hills CA USA; ^4^ Department of Cardiovascular Disease Creighton University School of Medicine, St. Joseph Hospital and Medical Center Phoenix AZ USA; ^5^ Cedars Sinai Medical Center California Los Angeles USA; ^6^ Cardiac Primary Prevention Research Center, Cardiovascular Diseases Research Institute Tehran University of Medical Sciences Tehran Iran; ^7^ Yale School of Medicine Yale University New Haven CT USA; ^8^ Department of Cardiology Medstar Union Memorial Hospital Baltimore MD USA; ^9^ Department of Cardiology Mayo Clinic School of Medicine Rochester MN USA

**Keywords:** aortic stenosis, balloon‐expandable valves, meta‐analysis, self‐expanding valves, TAVR, transcatheter aortic valve replacement

## Abstract

**Background:**

Comparisons of outcomes after transcatheter aortic valve replacement with balloon‐expandable (BEV) versus self‐expanding (SEV) valves are limited.

**Hypothesis:**

This study aimed to compare clinical and hemodynamic outcomes of BEV and SEV at short‐term (30 days), midterm (1 year), and long‐term (> 1 year) endpoints.

**Methods:**

PubMed, Embase, Scopus, and Cochrane Library databases were searched until July 2024 for randomized controlled trials. Random‐effect model (DerSimonian–Laird method) was used to pool the risk ratios (RR), mean differences, and 95% confidence intervals (CI).

**Results:**

A total of 10 studies comprising 4325 patients (2295 BEV, 2030 SEV) were included. In short‐term, cardiovascular (RR: 0.56, 95% CI: 0.36–0.87) and all‐cause mortality (RR: 0.54, 95% CI: 0.35–0.81) were lower in the BEV group. Risk of moderate to severe paravalvular leak (PVL) was lower among BEV patients in short‐term (RR: 0.28, 95% CI: 0.17–0.49) and long‐term (RR: 0.28, 95% CI: 0.1–0.79). A limited number of studies showed a greater risk of clinical valve thrombosis on BEV in midterm and long‐term. The need for permanent pacemaker implantation was lower in BEV at both short‐term (RR: 0.56, 95% CI: 0.37–0.87), and midterm (RR: 0.78, 95% CI: 0.64–0.94). The SEV group had a larger effective orifice area with lower mean transvalvular pressure gradient at all endpoints.

**Conclusions:**

BEV is associated with reduced risk of clinical outcomes in short‐term; however, most differences diminish in longer evaluations, except for moderate to severe PVL, which remains elevated for SEV. SEVs had better hemodynamic results and lower risk of clinical valve thrombosis.

## Summary

- In short‐term evaluations post‐TAVR, patients with BEV experienced better survival outcomes, but no difference was noted in longer evaluations.
- The need for permanent pacemaker implantation was also lower in BEV group at short‐term and midterm follow‐up periods.
- The SEVs had a larger effective orifice area with lower mean transvalvular pressure gradient even at long‐term endpoints post‐TAVR.

## Graphical abstract

By combining data from 10 studies comprising 4325 patients, this study found that balloon‐expandable valves were associated with a reduced risk of adverse clinical outcomes in the short term; which mainly disappeared in longer evaluations. Conversely, self‐expanding valves presented better hemodynamic performance.

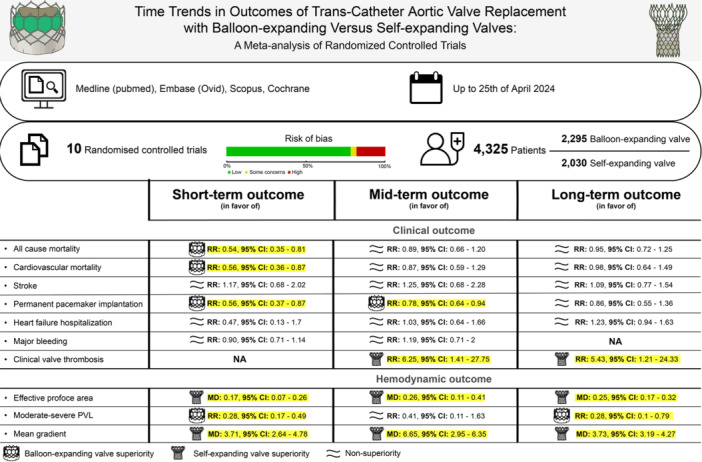

**Abbreviations**
BEV: balloon‐expandable valves
EOA: effective orifice area
PPI: permanent pacemaker implantation
PVL: paravalvular leak
RCT: randomized controlled trials
SEV: self‐expanding valves
TAVR: transcatheter aortic valve replacement

## 1 Introduction

Multiple studies have demonstrated transcatheter aortic valve replacement (TAVR) as an effective alternate to surgical aortic valve replacement for the treatment of aortic stenosis by offering less invasive procedure, non‐inferior clinical outcomes, and comparable hospitalization and follow‐up costs [1].

There are two major types of transcatheter heart valves used for TAVR, namely balloon‐expandable valves (BEV) and self‐expanding valves (SEV). Current guidelines do not provide clear recommendations on the optimal valve type [2, 3]. Previous meta‐analyses have shed some light on differences in outcomes of the two valve types [4, 5, 6, 7, 8], but their findings predominantly relied on observational studies, and long‐term outcomes were not available for comparison.

To address these gaps, we conducted a systematic review and meta‐analysis of randomized controlled trials (RCTs) to evaluate differences in short‐term, midterm, and long‐term clinical outcomes and hemodynamic performance of BEV and SEV in patients undergoing TAVR.

## 2 Materials and Methods

This study was conducted following the guidelines outlined in the Preferred Reporting Items for Systematic Reviews and Meta‐Analyses (PRISMA), as provided in Supporting Information S1: Table S1 [9]. The protocol of the study was registered in the International Prospective Register for Systematic Reviews (PROSPERO) (identification number: CRD42024541236).

### 2.1 Literature Search Strategy

Articles were identified through searches of Embase, PubMed, Scopus, and Cochrane Library from database inception to July 20, 2024. We also searched ClinicalTrials. gov for eligible published trials. Gray literature was searched using Google Scholar, with the first 100 links (sorted by relevance) evaluated against the inclusion criteria. No limitations were imposed in our searches. A meticulous hand search of the references in the included papers and relevant review articles was also conducted. Additionally, to ensure a comprehensive literature search, forward citation chaining was performed, which involved manually reviewing the literature that cited the included articles and relevant reviews in Scopus database. Search strategy of the databases are listed in Supporting Information S1: Table S2.

### 2.2 Study Selection and Eligibility Criteria

We included RCTs comparing clinical and hemodynamic outcomes of available BEVs against available SEVs in patients with aortic stenosis who underwent TAVR. Studies were excluded if they were review articles, lacked RCT design, were conducted on animal models, compared TAVR against surgical aortic valve replacement, compared BEV or SEV against mechanical valves, did not have available full text or lacked substantial information regarding the safety and efficacy of valve types.

The results of a systematic search were imported into the Rayyan web application [10]. Two reviewers (H.R. and S.M.N.) undertook an initial screening of titles and abstracts independently, with discrepancies being resolved by discussion with a third investigator (A.S.M.). For potentially relevant studies, full‐text was obtained and two investigators (S.P. and F.S.) independently assessed their eligibility. Disagreements about study inclusion were resolved by consensus with a third author (S.S.).

### 2.3 Risk of Bias and Quality Assessment

The risk of bias for each study was assessed by two independent reviewers (P.B. and N.S.H.) using Cochrane risk‐of‐bias tool for randomized trials version 2 (RoB 2) [11]. Moreover, the quality of evidence for each outcome parameter was assessed using the Grading of Recommendations, Assessment, Development, and Evaluations (GRADE) framework [12]. Agreement between the two observers was tested and disagreement was resolved by consensus.

### 2.4 Data Extraction

Two reviewers (P.B. and N.S.M.) independently collected the data from the included studies and recorded it in a predesigned Excel spreadsheet. A third reviewer (K.T.) cross‐checked the extracted data to identify any discrepancies. The required information was extracted from each eligible study across short‐term (30 days), midterm (1 year), and long‐term (> 1 year) endpoints as provided in Supporting Information S1: Table S3.

### 2.5 Statistical Analyses

Continuous variables were reported as means ± standard deviation or median (interquartile range), while categorical data were presented as numbers. Using random‐effect models, we estimated the pooled risk ratios (RRs) for binary outcomes and mean differences (MD) for continuous outcomes with their 95% confidence interval (CI) using the Mantel–Haenszel method. A continuity correction of 0.5 was applied for studies without event in one of the valve groups. The heterogeneity was measured using Cochran Q and I^2^ statistics. In analyses where I^2^ > 50% was observed, sensitivity analyses were conducted by excluding each study one by one to test the robustness of the pooled estimates and to evaluate the effects of each selected study on the overall results of the meta‐analysis. Moreover, potential heterogeneity factors were explored for all analyses by prespecified subgroup analyses including low surgical risk patients, small aortic annulus patients, old generation SEV (CoreValve) or newer SEVs (Evolut, Acurate, or Portico), or SEV brand used. The subgroup analysis for BEV generations was hindered by the limited number of studies used the older generations [13]. *p*‐values were 2‐sided, and values < 0.05 were considered significant. All statistical analyses were conducted using R Programming language (R for Windows, version 4.1.3, Vienna, Austria) and R Studio version 1.1.463 (Posit PBC, Boston, MA, United States) utilizing the “dmetar” and “meta” statistical packages [14].

## 3 Results

**FIGURE 1:**
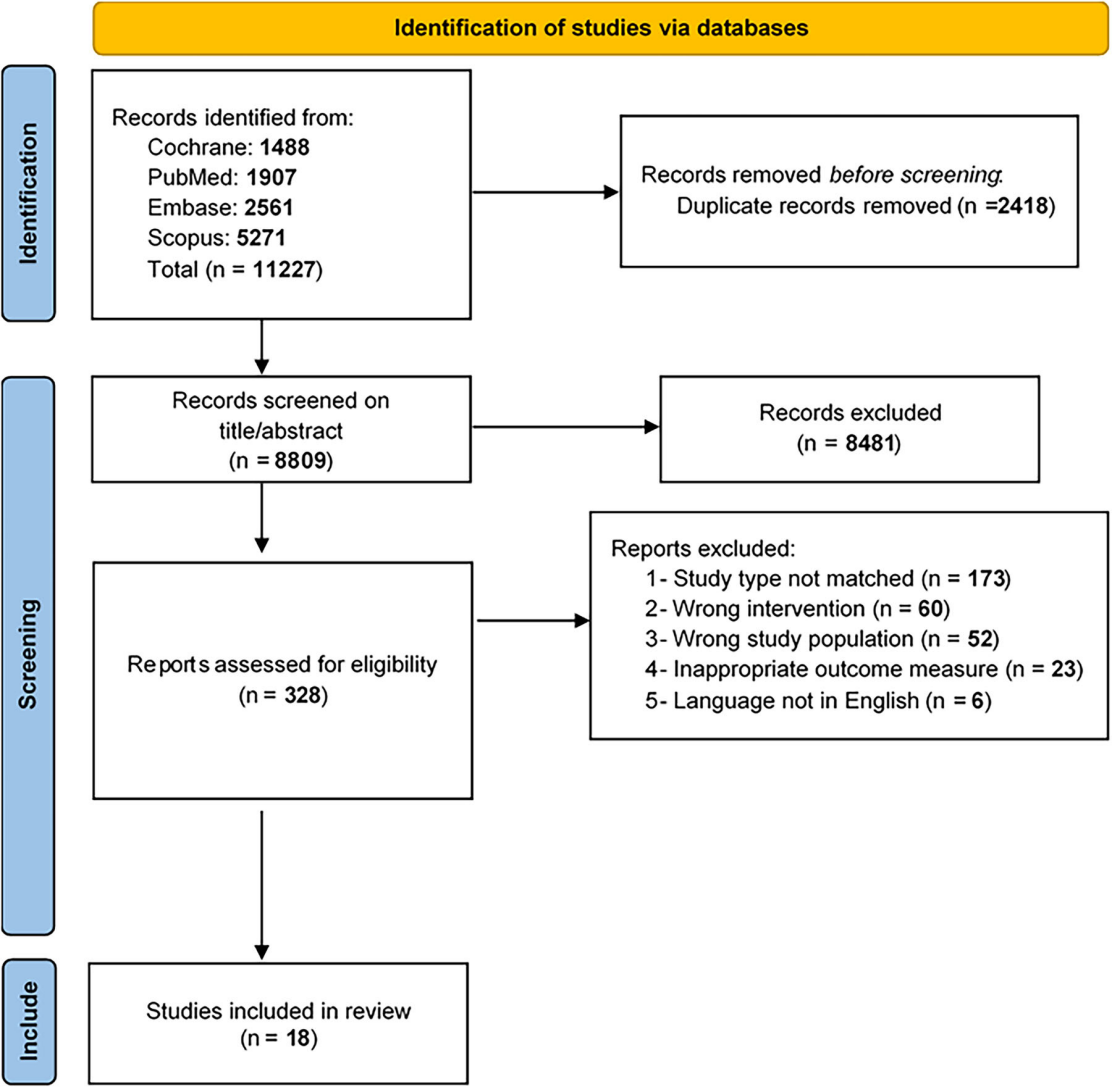
Study flow chart.

Initially, 11,227 studies were identified through our primary search. After removal of duplicates, 8809 studies were subjected to title and abstract screening. Subsequently, 328 studies were assessed for full‐text screening and eligibility, and 18 studies were included in our meta‐analysis (Figure 1). These studies, from 10 RCTs, comprised a total of 4325 patients undergoing TAVR (2295 BEV, 2030 SEV) [13, 15, 16, 17, 18, 19, 20, 21, 22, 23, 24, 25, 26, 27, 28, 29, 30, 31]. The complete characteristics of the included studies are available in Table 1 and Supporting Information S1: Table S4.

**TABLE 1:** Characteristics of included studies.

Clinical trial	Studies published	Follow up	Region	Surgical risk*	Small annulus	Valve type	Valve brand	Sample size	Demographic	
									Age, year (mean ± SD)	Male (%)
BRAVO 3	Linke 2017 [13]	30 days	Europe and North America	High	No	SEV	Medtronic CoreValve	282	82.9 ± 6.1	49
						BEV	Edwards SAPIEN	500	81.8 ± 6.7	55
CHOICE	Abdel‐Wahab 2014 [15]	30 days	Europe	High	No	SEV	Medtronic CoreValve	120	79.6 ± 15.8	28.3
	Abdel‐Wahab 2015 [16]	1 year								
	Abdelghani 2018 [17]	30 days				BEV	Edwards Sapien XT	121	81.9 ± 6.7	43
	Abdel‐Wahab 2020 [21]	5 years								
ELECT	Kooistra 2020 [22]	30 days–1 year	Europe	High	No	SEV	Medtronic CoreValve	27	81 ± 7	52
						BEV	Edwards SAPIEN 3	29	77 ± 11	48
LYTEN	Rodés‐Cabau 2022 [25]	30 days	Europe and North America	Low–intermediate–high	Yes	SEV	Medtronic Evolut R/Pro/Pro +	53	80 ± 6	47
	Nuche 2023 [28]	1 year				BEV	Edwards SAPIEN 3/ULTRA	49	79 ± 6	59
PORTICO IDE	Makkar 2020 [19]	30 days–1 year–2 years	North America and Australia	High	No	SEV	Portico	375	83 ± 7.6	48
						BEV	Edwards SAPIEN 3	206	83.5 ± 7.4	48.1
SOLVE‐TAVI	Thiele 2020 [20]	30 days	Europe	Intermediate–high	No	SEV	Medtronic Evolut R	219	81.7 ± 5.3	48
						BEV	Edwards SAPIEN 3	219	81.5 ± 5.7	50
	Feistritzer 2021 [23]	1 year								
	Farhan 2022 [27]	30 days–1 year								
SCOPE 1	Lanz 2019 [18]	30 days	Europe	High	No	SEV	ACURATE neo	372	82.6 ± 4.3	41.4
	Kim 2020 [24]	1 year				BEV	Edwards SAPIEN 3	367	83 ± 3.9	45
	Lanz 2023 [29]	3 years								
_	Elnaggar 2022 [26]	1 week	Europe	Low–intermediate–high	Yes	SEV	Medtronic Evolut PRO	51	82.6 ± 6.4	54.9
						BEV	Edwards SAPIEN 3	59	81.2 ± 5.8	59.3
SMART	Herrmann 2024 [31]	30 days–1 year	Europe, North America and Middle East	Low–intermediate–high	Yes	SEV	Medtronic Evolut R + Evolut PRO + Evolut FX	355	80.1 ± 6.3	12.1
						BEV	Edwards SAPIEN 3 + SAPIEN 3 Ultra	361	80.3 ± 6.1	14.4
LANDMARK	Baumbach 2024 [30]	30 days	Europe, Australia and South America	Low–intermediate–high	Yes	SEV	Medtronic Evolut	192	NA	NA
						BEV	Myval	384	80·0 ± 5·7	50

Abbreviations: BEV, balloon‐expanding valves; SEV, self‐expanding valves.

* Based on the Society of Thoracic Surgeons Predicted Risk of Mortality.

In the risk of bias assessment, seven RCTs were assessed as “low” risk of bias, two RCTs had “some concerns,” and one RCT assessed as “high” risk of bias (Figures S1 and S2). The quality of evidence for most of the outcomes was rated as “moderate,” primarily due to the limited number of studies and the frequency of observed outcomes, as provided in Supporting Information S1: Table S5.

### 3.1 Clinical Outcomes

**FIGURE 2:**
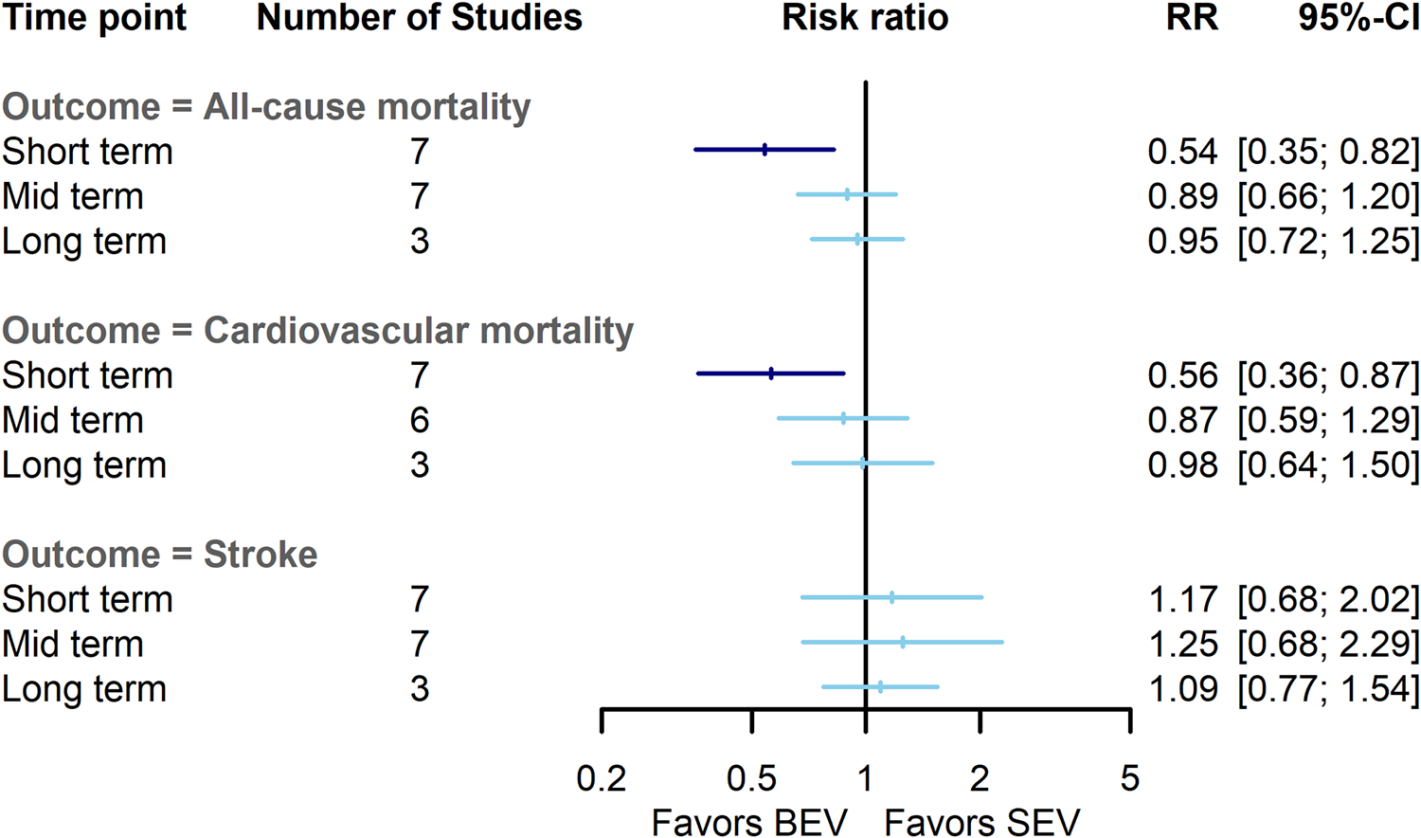
Comparison of balloon‐expandable valves with self‐expanding valves for all‐cause mortality, cardiovascular mortality, and stroke at short‐term, midterm, and long‐term. BEV, balloon‐expandable valve; CI, confidence interval; RR, risk ratio; SEV, self‐expanding valve.

At short‐term follow‐up, BEV patients experienced a lower risk of all‐cause mortality compared to SEV (RR = 0.54, 95% CI: 0.35–0.81, *p*‐value: 0.003) with no heterogeneity between studies (I^2^: 0%, Q: 5.4, *p*‐value: 0.49). The risk of all‐cause mortality became comparable between BEV and SEV at midterm (RR = 0.89, 95% CI: 0.66–1.20, *p*‐value: 0.43) and long‐term follow‐ups (RR = 0.95, 95% CI: 0.79–1.25, *p*‐value: 0.71), but with moderate (I^2^: 34%, Q: 9, *p*‐value: 0.17) and high heterogeneity (I^2^: 61%, Q: 5, *p*‐value: 0.07), respectively (Figures 2 and S3A). Sensitivity analysis demonstrated consistent results after excluding studies one‐by‐one at long term outcomes.

The risk of cardiovascular mortality was lower in the BEV group at short‐term follow‐up (RR = 0.56, 95% CI: 0.36–0.87, *p*‐value: 0.01) with no heterogeneity (I^2^: 0%, Q: 5.6, *p*‐value: 0.46). Nevertheless, this risk did not differ between BEV and SEV at midterm (RR = 0.87, 95% CI = 0.59–1.29, *p*‐value: 0.49) and long‐term follow‐ups (RR = 0.98, 95% CI: 0.64–1.49, *p*‐value: 0.93), with low heterogeneity at midterm endpoint (I^2^: 22%, Q: 6.3, *p*‐value: 0.27), and high heterogeneity in log‐term (I^2^: 66%, Q: 5.8, *p*‐value: 0.05) (Figures 2 and S3B). These findings were robust after performing sensitivity analysis in long‐term outcomes.

No significant difference for stroke was observed between BEV and SEV during short‐term (RR = 1.17, 95% CI = 0.68–2.02, *p*‐value: 0.57), midterm (RR = 1.25, 95% CI = 0.68–2.28, *p*‐value: 0.47), and long‐term (RR = 1.09, 95% CI = 0.77–1.54, *p*‐value: 0.61). The heterogeneity was moderate to low in short‐term (I^2^: 37%, Q: 9.5, *p*‐value: 0.14) and long‐term endpoints (I^2^: 0%, Q: 0.4, *p*‐value: 0.79). Yet, there was high heterogeneity between studies at midterm follow‐up (I^2^: 53%, Q: 21.8, *p*‐value: 0.04) (Figures 2 and S3C). In sensitivity analysis regarding midterm outcomes, the removal of no single study significantly changed the results. Furthermore, concerning stroke subtypes (disabling and nondisabling), the risk remained similar for both subgroups at all follow‐up intervals (Figure S4).

**FIGURE 3:**
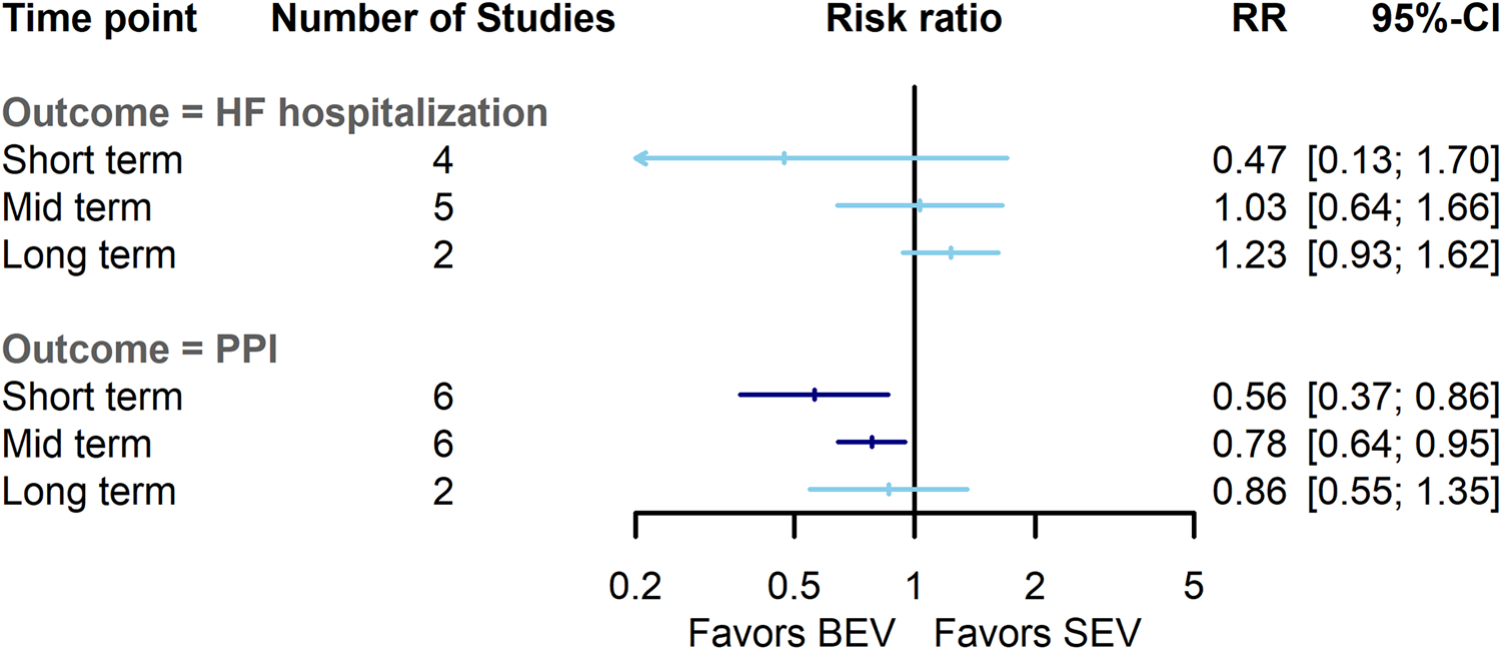
Comparison of balloon‐expandable valves with self‐expanding valves for heart failure hospitalization, and permanent pacemaker implantation at short‐term, midterm, and long‐term. BEV, balloon‐expandable valve; CI, confidence interval; HF, heart failure; PPI, permanent pacemaker implantation; RR, risk ratio; SEV, self‐expanding valve.

**FIGURE 4:**
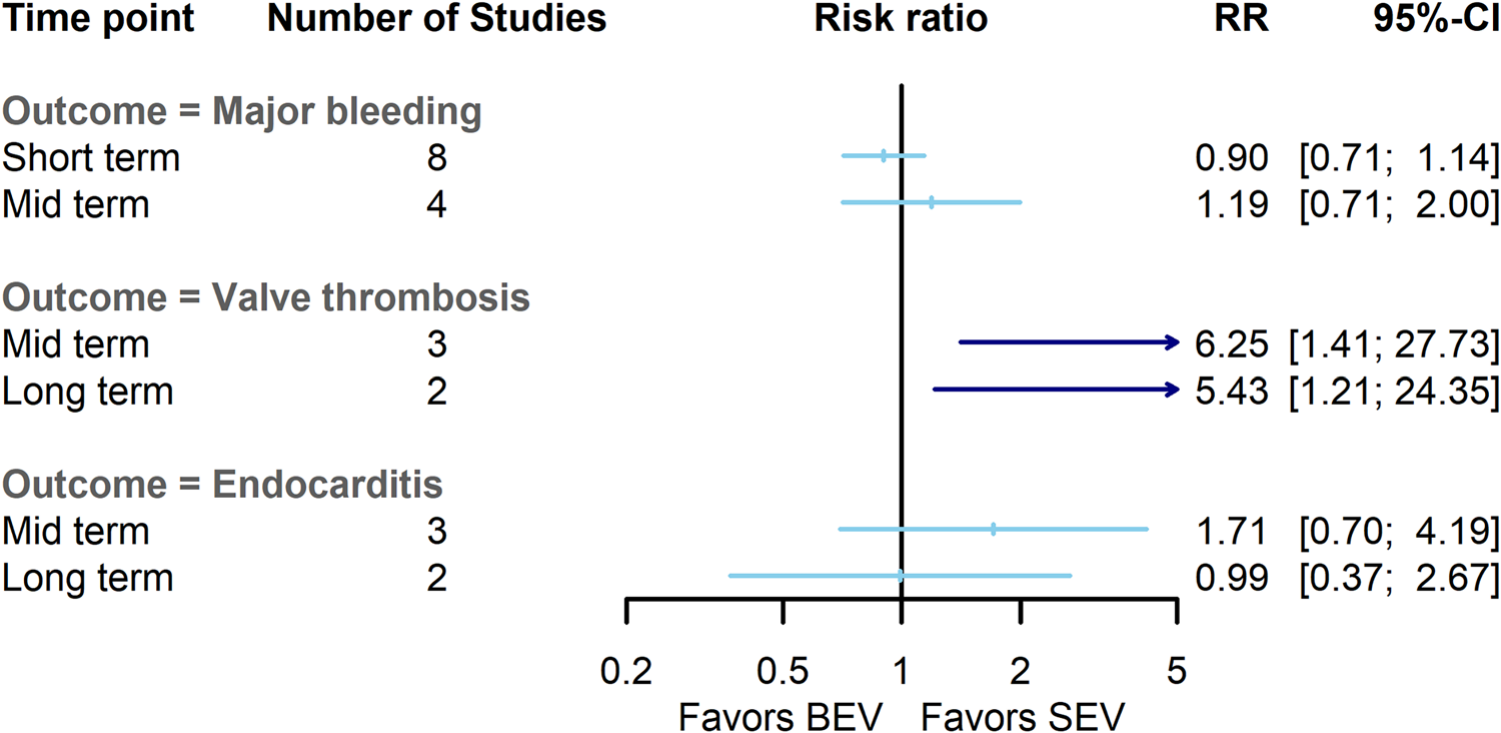
Comparison of balloon‐expandable valves with self‐expanding valves for major bleeding event, clinical valve thrombosis, and endocarditis at short‐term, midterm, and long‐term. BEV, balloon‐expandable valve; CI, confidence interval; RR, risk ratio; SEV, self‐expanding valve.

Notably, BEV patients had a lower risk of permanent pacemaker implantation (PPI) compared to SEV at short‐term (RR = 0.56, 95% CI = 0.37–0.87, *p*‐value: 0.008) and midterm (RR = 0.78, 95% CI = 0.64–0.94, *p*‐value: 0.01) follow‐ups. Nonetheless, this difference converged at long‐term follow‐up and became comparable between BEV and SEV (RR = 0.86, 95% CI = 0.55–1.36, *p*‐value: 0.52). The heterogeneity between studies was low in midterm assessment (I^2^: 0%, Q: 4, *p*‐value: 0.53), however, the heterogeneity a short‐term (I^2^: 74%, Q: 19.3, *p*‐value: 0.001) and long‐term (I^2^: 64%, Q: 2.7, *p*‐value: 0.09) outcomes were reported high (Figures 3 and S5B). None of the sensitivity analysis substantially altered lower risk of PPI in BEV group in short‐term endpoints. BEV was associated with an increased risk of valve thrombosis compared to SEV in midterm (RR = 6.25, 95% CI = 1.41–27.75, *p*‐value: 0.01) and long‐term (RR = 5.43, 95% CI = 1.21–24.33, *p*‐value: 0.02), with low heterogeneity at both endpoints (I^2^: 0%, Q: 0.1, *p*‐value: 0.94, and I^2^: 0%, Q: 0.02, *p*‐value: 0.88) (Figures 4 and S6B).

As for the other clinical outcomes, the risks of acute kidney injury, new atrial fibrillation, heart failure hospitalization, major bleeding, major cardiovascular events, and endocarditis were similar between two valve types (Figures 3 and 4) (Figures S5A, S6A, S7 and S8). The results of subgroup analysis based on the surgical risk of the patient, anatomical characteristics of the aortic annulus, and SEV brands and generations are presented in the Data S1.

### 3.2 Hemodynamic Outcomes

**FIGURE 5:**
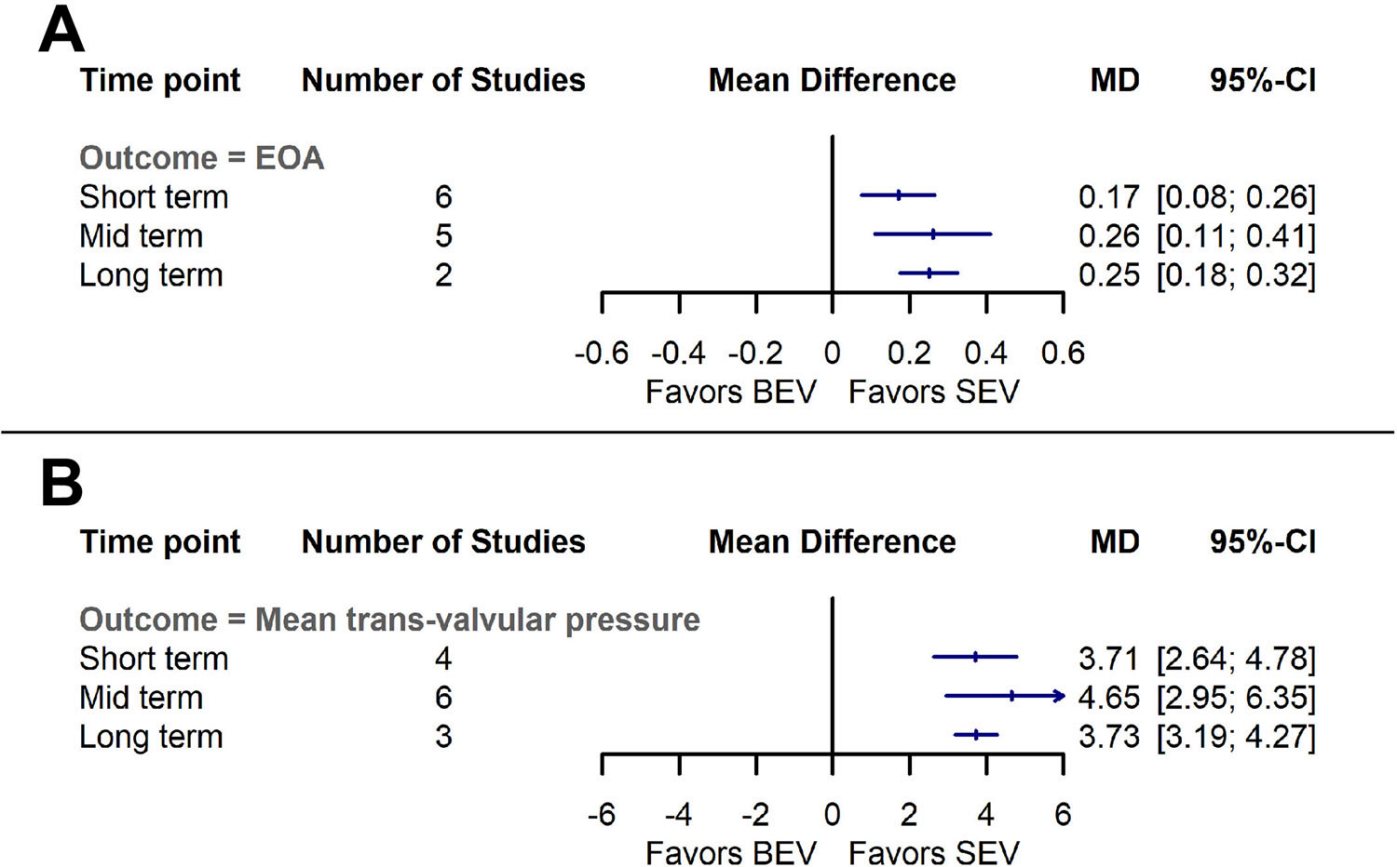
Comparison of balloon‐expandable valves with self‐expanding valves for (A) effective orifice area (cm^2^) (B) and mean transvalvular pressure gradient (mmHg). BEV, balloon‐expandable valve; CI, confidence interval; MD, mean difference; SEV, self‐expanding valve.

The results demonstrated a significantly larger effective orifice area (EOA) in the SEV group in short‐term (MD = 0.16, 95% CI: 0.07–0.25 cm^2^, *p*‐value < 0.001), midterm (MD = 0.26, 95% CI: 0.11–0.41 cm^2^, *p*‐value < 0.001), and long‐term evaluations (MD = 0.24, 95% CI: 0.17–0.32 cm^2^, *p*‐value < 0.001) (Figures 5A and S9A). The heterogeneity was low in long‐term evaluations (I^2^: 0%, Q: 0.7, *p*‐value: 0.4), but was high in short‐term (I^2^: 70%, Q: 17, *p*‐value: 0.004) and midterm (I^2^: 89%, Q: 37, *p*‐value < 0.001) follow‐ups. However, the leave‐one‐out analysis demonstrated that after the exclusion of Baumbach et al. study [30], the heterogeneity in short‐term outcome decreased considerably (I^2^: 19%, Q: 4.9, *p*‐value: 0.2). of note, by excluding Lanz et al. [18] or Makkar et al. [19] studies, the EOA became comparable between both groups in short‐term follow‐up (MD = 0.12, 95% CI: −0.01–0.26 cm^2^, and MD = 0.12, 95% CI: −0.01–0.26 cm^2^, respectively). The significantly higher EOA in the SEV group in midterm follow‐ups was consistent by excluding studies through sensitivity analysis.

The result from RCTs suggest that mean transvalvular pressure gradient was significantly lower in the SEV cases in all study periods, including short‐term (MD = −3.71, 95% CI: −4.78 to −2.64 mmHg, *p*‐value < 0.001), midterm (MD = −4.65, 95% CI: −6.35 to −2.95 mmHg, *p*‐value < 0.001), and long‐term (MD = −3.73, 95% CI: −4.27 to −3.19 mmHg, *p*‐value < 0.001) follow‐ups (Figures 5B and S9B). The heterogeneity was reported high in short‐term (I^2^: 82%, Q: 16, *p*‐value: 0.008) and midterm (I^2^: 92%, Q: 5, *p*‐value < 0.001) outcomes. But the heterogeneity was low in long‐term results (I^2^: 10%, Q: 2, *p*‐value: 0.32). None of the sensitivity analysis substantially altered the outcomes in short‐term and midterm.

**FIGURE 6:**
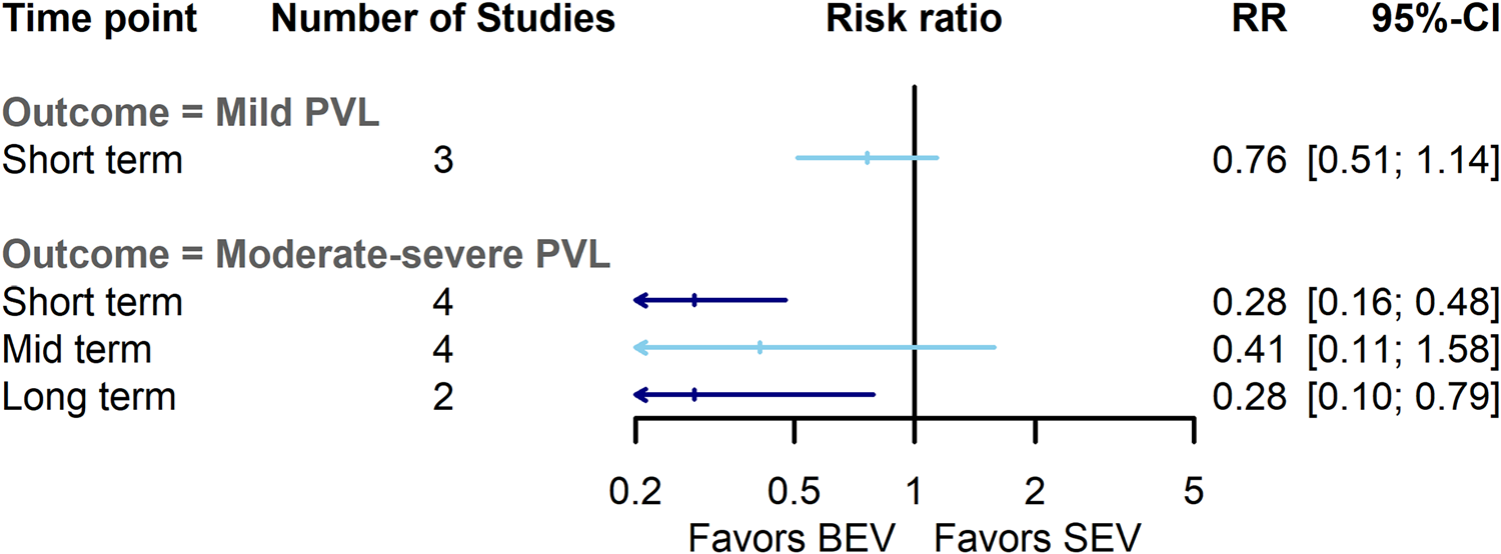
Comparison of balloon‐expandable valves with self‐expanding valves for mild and moderate–severe paravalvular leak. BEV, balloon‐expandable valve; CI, confidence interval; PVL, paravalvular leak; RR, risk ratio; SEV, self‐expanding valve.

The risk of moderate to severe paravalvular leak (PVL) was significantly lower in the BEV group in short‐term (RR = 0.28, 95% CI: 0.17–0.49, *p*‐value < 0.001) and long‐term (RR = 0.28, 95% CI: 0.10–0.79, *p*‐value: 0.01) follow‐ups with low heterogeneity (I^2^: 0%, Q: 1.4, *p*‐value: 0.69; and I^2^: 0%, Q: 0.45, *p*‐value: 0.5, respectively). However, a comparable risk between the two groups was observed through midterm (RR = 0.41, 95% CI: 0.11–1.63, *p*‐value: 0.2) with high heterogeneity (I^2^: 65%, Q: 8, *p*‐value: 0.03) (Figures 6 and S9C). The sensitivity analysis unveiled a lower risk of moderate to severe PVL incidence in BEV cases through midterm follow‐up (RR = 0.27, 95% CI: 0.08–0.95) with lower heterogeneity (I^2^ = 61%) by excluding Hermann et al. study [31]. Concerning mild PVL, a comparable risk was demonstrated between the two groups in short‐term follow‐up (Figures 6 and S10), which was also consistent in the sensitivity analysis.

The results of the subgroup analysis, based on patient surgical risk, anatomical characteristics of the aortic annulus, and SEV brand and generations, are presented in the Data S1, along with the procedural characteristics results.

## 4 Discussion

The evidence from currently available RCTs suggests the following outcomes: (1) In short‐term, the risk of all‐cause mortality and cardiovascular mortality was lower in the BEV group, but no difference was noted between the two treatment modalities in extended follow‐ups. (2) The risk of moderate to severe PVL was lower among BEV patients during both short‐term and long‐term endpoints. (3) SEV patients experienced reduced occurrences of clinical valve thrombosis in midterm and long‐term follow‐ups. (4) The need for PPI was lower in BEV patients in short‐term and midterm but not in long‐term follow‐ups. (5) The SEV group had a larger EOA as well as a lower mean transvalvular pressure gradient through all endpoints. (Graphical Abstract).

### 4.1 Clinical Outcomes

This study demonstrated that BEV patients had reduced risk of all‐cause mortality and cardiovascular mortality in the short‐term period despite the comparable results in extended evaluations. These findings updated the results of the previous meta‐analyses demonstrating better short‐term survival in BEV patients [6, 32]. The findings also align with the FRANCE‐TAVI registry, which indicated that SEV patients had a higher risk of mortality at the 3‐months endpoint [33]. The low heterogeneity in our analyses indicated that most of the included RCTs agreed on lower all‐cause and cardiovascular mortality in short‐term among BEV patients including the recent SMART trial [31]. The observed short‐term survival benefit in the BEV group can be attributed to the differences in the procedural characteristics between the two valve types like quicker deployment modes and reduced need for implantation of more than one valve per procedure. Larger RCTs are required to draw stronger conclusions as the rationale behind the better short‐term survival in the BEV group is still unclear.

Our results suggest that the risk of stroke was comparable between the two treatment modalities through all follow‐up endpoints, which was in line with previous findings [5, 6]. Nevertheless, other reviews contradicted our outcomes by showing that the risk of stroke was lower in BEV patients in short‐term evaluations [7, 8]. Their results, however, were based on propensity score matched studies, which are prone to bias from confounders. Notably, the risk of clinical valve thrombosis was higher in the BEV group in our study in midterm and long‐term periods. These results are consistent with the evidence in BEV patients in PANTER trial compared to SEV patients in Evolut low risk trial [34, 35]. Moreover, subclinical valve thrombosis was reported to be higher in BEV patients in previous trials [36]. Nevertheless, only a limited number of studies evaluated the risk of valve thrombosis in BEV and SEV patients in our analyses. Previous articles demonstrated an association between valve thrombosis and future cerebrovascular events [37]. Some studies have found that oral anticoagulation therapy can be more effective than antiplatelet treatments in mitigating valve thrombosis progression, but the increased risk of bleeding may offset these benefits [37]. Further research is warranted to evaluate the risk of valve thrombosis between the two valve types and to assess the impact of anticoagulation therapy, especially in younger patients with lower surgical risk for improved durability.

This study also demonstrated that the risk of PPI was lower in the BEV group both in short‐term and midterm evaluations like previous studies [5, 6, 8]. The differences between the outcomes may arise from the variations in the stent design, the optimal depth of valve implantation, and the radial force exerted on the conducting system tissue [7]. Interestingly, the risk of PPI was comparable between the two valve types in long‐term evaluations in our study. Despite the higher risk of conduction disturbances in SEV patients, nearly half of their conduction disturbances resolved over time without requirement of PPI, which may partly explain the comparable outcomes in longer follow‐ups [38]. Further research is required to assess the risk of PPI between the two treatment modalities in long‐term evaluations.

### 4.2 Hemodynamic Outcomes

Another major concern with the TAVR procedure is the relatively small postprocedural EOA and high transvalvular pressure gradient. Previous studies reported that SEV was associated with a larger postprocedural EOA and lower transvalvular gradient than BEV [4, 7]. The current meta‐analysis supported these findings. This advantage is likely due to the supra‐annular position of the SEV leaflets, compared with BEVs with an intra‐annular design, which allows for lower resistance to left ventricular outflow and gradients [7]. Notably, the supra‐annular position of SEV valves is crucial for better hemodynamic outcomes but is not the only factor. Previous meta‐analysis demonstrated that intra‐annular SEVs like Portico have better hemodynamic performance compared supra‐annular ones like Acurate [39]. Therefore, other factors like large cell area at the annulus section of the stent frame and the method of leaflet suturing to minimize tissue interface, should also be considered.

Smaller EOA and higher mean transvalvular pressure gradient can negatively impact the patients' quality of life and accelerate valve degeneration [31]. However, the suboptimal hemodynamic performance in BEV group did not translate to worsened clinical outcomes in this study. Previous studies have demonstrated discordant results between catheterization and Doppler echocardiography in evaluating postprocedural transvalvular mean pressure gradients [28]. Doppler echocardiography often overestimates the mean transvalvular pressure gradients, especially in BEV patients [40] This calls into the need for further evaluations of postprocedural hemodynamic characteristics of TAVR valves especially using invasive methods.

This study also showed that the risk of moderate to severe PVL was lower in the BEV group both in short‐term and long‐term endpoints. The increased risk of moderate to severe PVL with SEV can be attributed to the lower radial strength of the nitinol frame used in SEV alongside their higher eccentricity index and decreased expansion. Moderate to severe PVL may be associated with higher short‐term and long‐term mortality after TAVR in aortic stenosis patients [20]. Nevertheless, our results indicated that the risk of mortality was comparable between the two valves in midterm and long‐term. These findings reinforce the fact that other unknown risk factors may also be associated with mortality which remains to be studied. The main findings of previous meta‐analyses are presented in Supporting Information S1: Table S6.

## 5 Limitations

There are some limitations that should be mentioned regarding the present systematic review and meta‐analysis. First, the included RCTs mostly lack sufficient power for evaluating some outcomes, leading to the loss of statistical significance in some subgroup and sensitivity analyses. Nevertheless, this meta‐analysis included more than twice as many RCTs as previous reports allowing for a more precise estimate of TAVR valve outcomes. More adequately powered RCTs are needed to compare the two TAVR modalities comprehensively. Second, despite conducting subgroup analyses, substantial heterogeneity persisted across some evaluations. This heterogeneity may be attributed to the varying characteristics of different SEV brands used in the studies. Only two RCTs employed SEVs other than CoreValve or Evolut [18, 19, 24, 29]. However, since only one BEV brand was compared against multiple iterations of SEVs from different manufacturers with various designs, the findings cannot be extrapolated to any specific SEV type. Additionally, the variations in design within the same SEV and BEV brands, as well as differences in diameters used for the included participants, could contribute to potential heterogeneity. However, due to the lack of patient‐level data, further analysis to account for these design differences was not possible. Third, tests for publication bias were not undertaken, as Egger's test and funnel plots are not suitable for analysis with fewer than 10 studies. Last, there were a limited number of RCTs evaluating the long‐term outcomes of TAVR. Further evaluations on long‐term outcomes of BEV and SEV are still required.

## 6 Conclusion

In the recent guidelines, TAVR has received a Class I, Level A recommendation for patients over 75‐years‐old patients and those considered high risk for surgery, with a strong emphasis on shared decision‐making between patients and the experts [2, 3]. The present meta‐analysis provided a comprehensive investigation to compare the outcome of two TAVR modalities. The results indicated that each valve type is associated with distinct advantages and disadvantages post‐TAVR. Therefore, the medical team should consider device characteristics besides the patient's condition to minimize the risk of complications.

## Ethics Statement

The authors have nothing to report.

## Conflicts of Interest

The authors declare no conflicts of interest.

## Supporting information


**Figure S1.** Summery bar plot for risk of bias assessment of the included studies.

**Figure S2.** Summery traffic light plot for risk of bias assessment of the included studies.

**Figure S3.** Comparison of balloon‐expandable valves with self‐expanding valves for (A) all‐cause mortality (B) cardiovascular mortality and (C) stroke at short‐term, midterm, and long‐term. BEV, balloon‐expandable valve; CI, confidence interval; SEV, self‐expanding valve; RR, Risk ratio.

**Figure S4.** Comparison of balloon‐expandable valves with self‐expanding valves for disabling and nondisabling stroke at short‐term, midterm, and long‐term. BEV, balloon‐expandable valve; CI, confidence interval; RR, risk ratio; SEV, self‐expanding valve.

**Figure S5.** Comparison of balloon‐expandable valves with self‐expanding valves for (A) heart failure hospitalization (B) and permanent pacemaker implantation at short‐term, midterm, and long‐term. BEV, balloon‐expandable valve; CI, confidence interval; RR, risk ratio; SEV, self‐expanding valve.

**Figure S6.** Comparison of balloon‐expandable valves with self‐expanding valves for (A) major bleeding event (B) and clinical valve thrombosis at short‐term, midterm, and long‐term. BEV, balloon‐expandable valve; CI, confidence interval; RR, risk ratio; SEV, self‐expanding valve.

**Figure S7.** Comparison of balloon‐expandable valves with self‐expanding valves for endocarditis at short‐term, and midterm. BEV, balloon‐expandable valve; CI, confidence interval; RR, risk ratio; SEV, self‐expanding valve.

**Figure S8.** Comparison of balloon‐expandable valves with self‐expanding valves for AKI, new atrial fibrillation, and major cardiovascular event at short‐term. AKI, acure kidney injury; BEV, balloon‐expandable valve; CI, confidence interval; RR, risk ratio; SEV, self‐expanding valve.

**Figure S9.** Comparison of balloon‐expandable valves with self‐expanding valves for effective orifice area (cm2) (B) mean transvalvular pressure gradient (mmHg) and (C) moderate to severe PVL at short‐term, mid‐term, and long‐term. BEV, balloon‐expandable valve; CI, confidence interval; MD, mean difference; PVL, paravalvular leak; SEV, self‐expanding valve.

**Figure S10.** Comparison of balloon‐expandable valves with self‐expanding valves for mild PVL at short‐term. BEV, balloon‐expandable valve; CI, confidence interval; PVL, paravalvular leak; RR, risk ratio; SEV, self‐expanding valve.

Supplementary Figures legends.

Supplementary Manuscript.

Supplementary Tables.

## Data Availability

The data that support the findings of this study are available from the corresponding author upon reasonable request.
