# Supplementary figures and images for: Short‐Term, Mid‐Term, and Long‐Term Outcomes of Transcatheter Aortic Valve Replacement With Balloon‐Expandable Versus Self‐Expanding Valves: A Meta‐Analysis of Randomized Controlled Trials
Source: Clin Cardiol. 2025 Apr 19;48(4):e70134. doi: 10.1002/clc.70134 (PMC12008748; doi:10.1002/clc.70134)

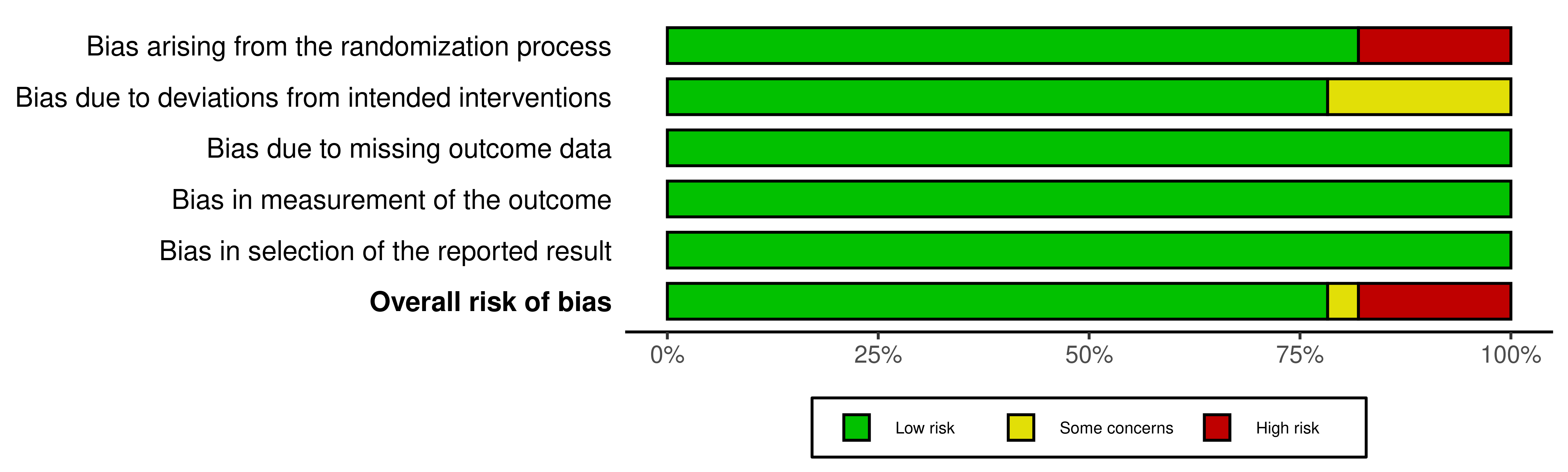

Supplement: Supplementary file 1 — Figure S1. Summery bar plot for risk of bias assessment of the included studies. [file CLC-48-e70134-s009.png]

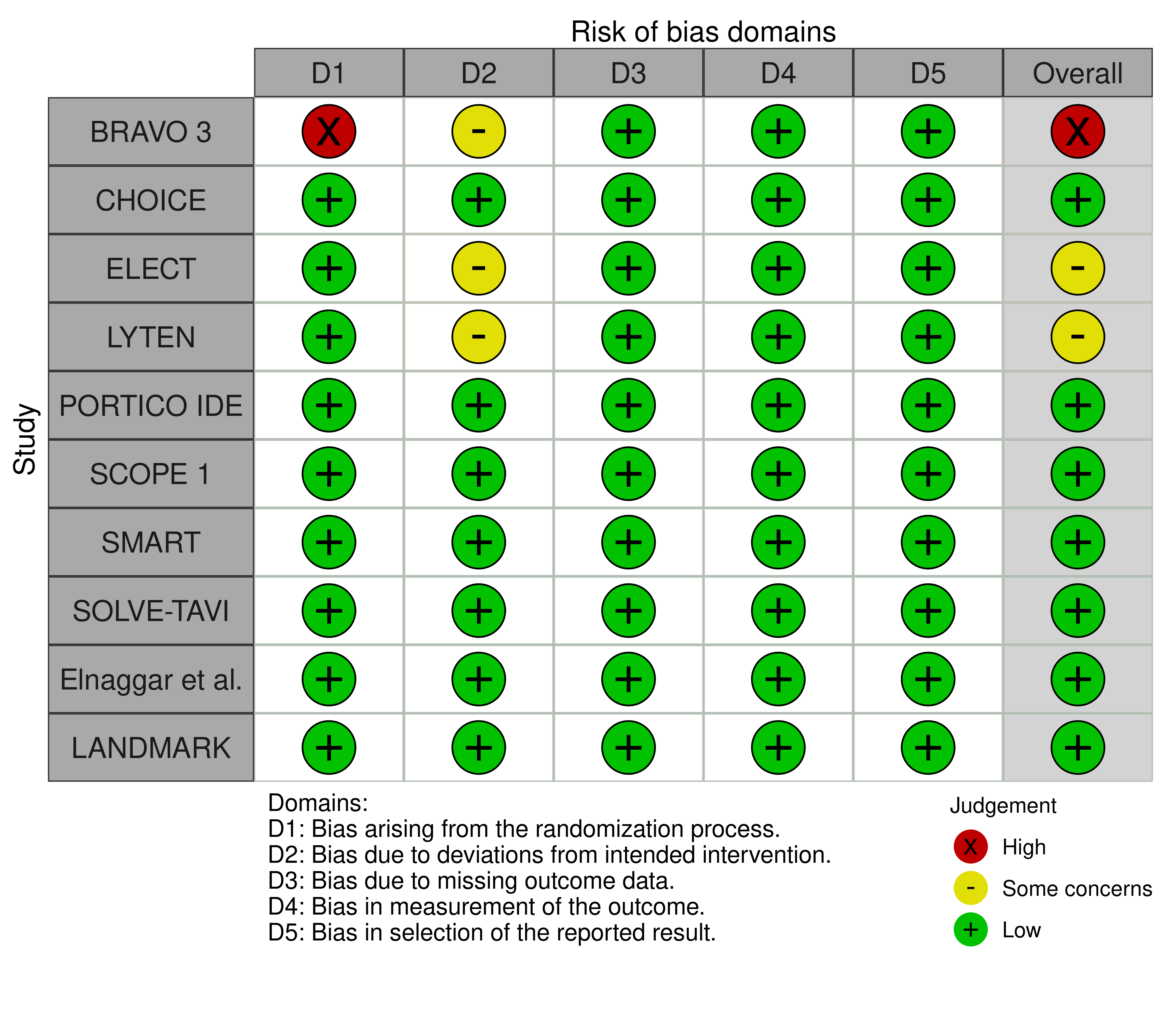

Supplement: Supplementary file 2 — Figure S2. Summery traffic light plot for risk of bias assessment of the included studies. [file CLC-48-e70134-s003.png]

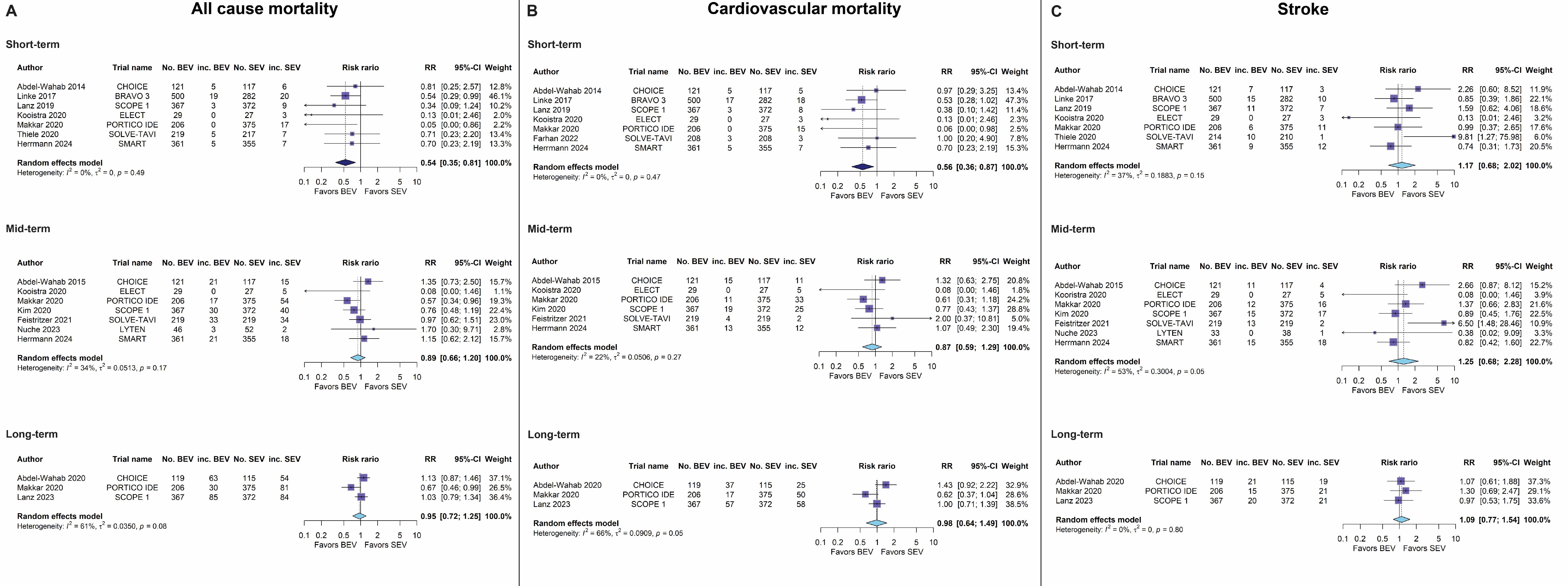

Supplement: Supplementary file 3 — Figure S3. Comparison of balloon‐expandable valves with self‐expanding valves for (A) all‐cause mortality (B) cardiovascular mortality and (C) stroke at short‐term, midterm, and long‐term. BEV, balloon‐expandable valve; CI, confidence interval; SEV, self‐expanding valve; RR, Risk ratio. [file CLC-48-e70134-s004.jpg]

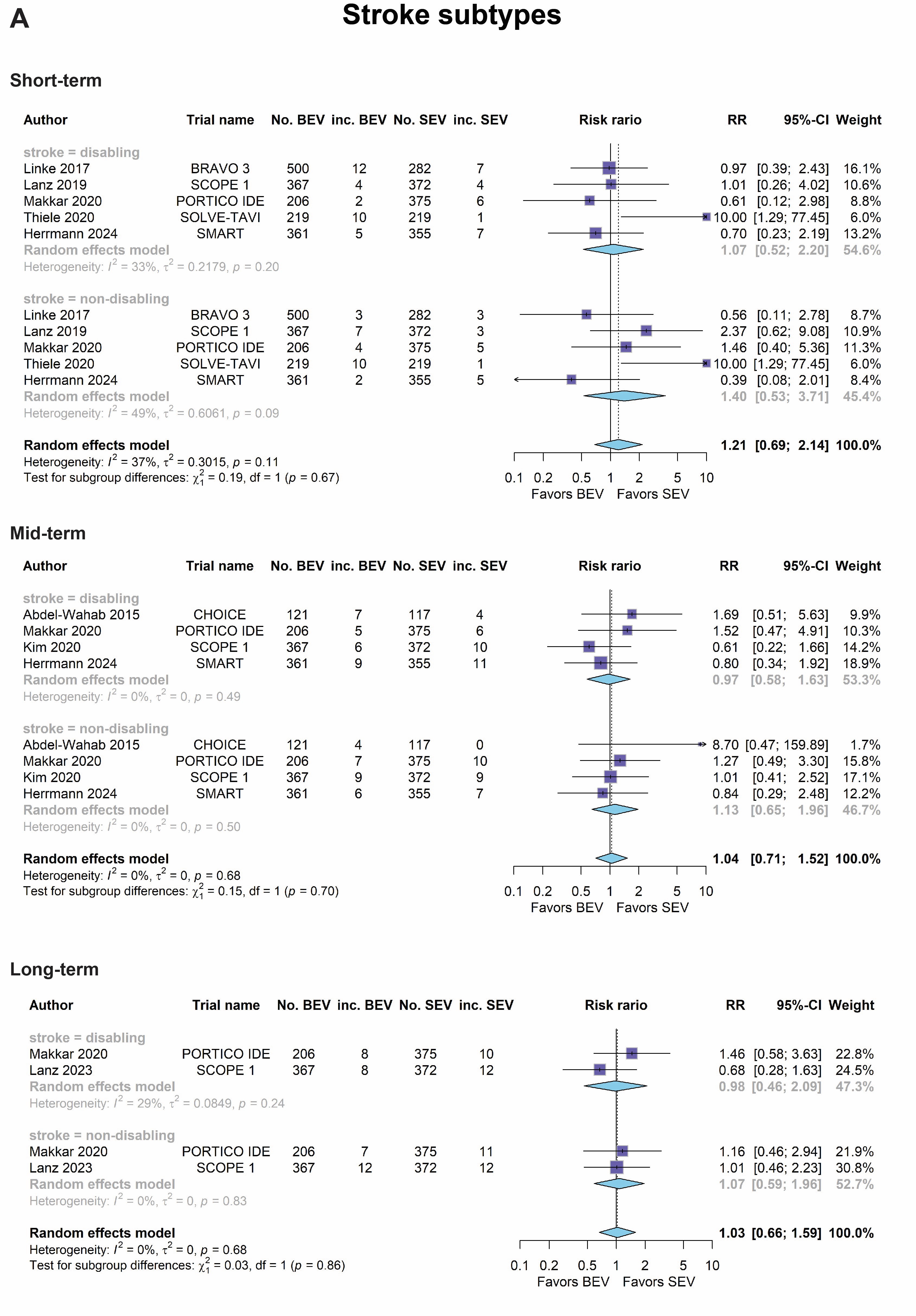

Supplement: Supplementary file 4 — Figure S4. Comparison of balloon‐expandable valves with self‐expanding valves for disabling and nondisabling stroke at short‐term, midterm, and long‐term. BEV, balloon‐expandable valve; CI, confidence interval; RR, risk ratio; SEV, self‐expanding valve. [file CLC-48-e70134-s010.jpg]

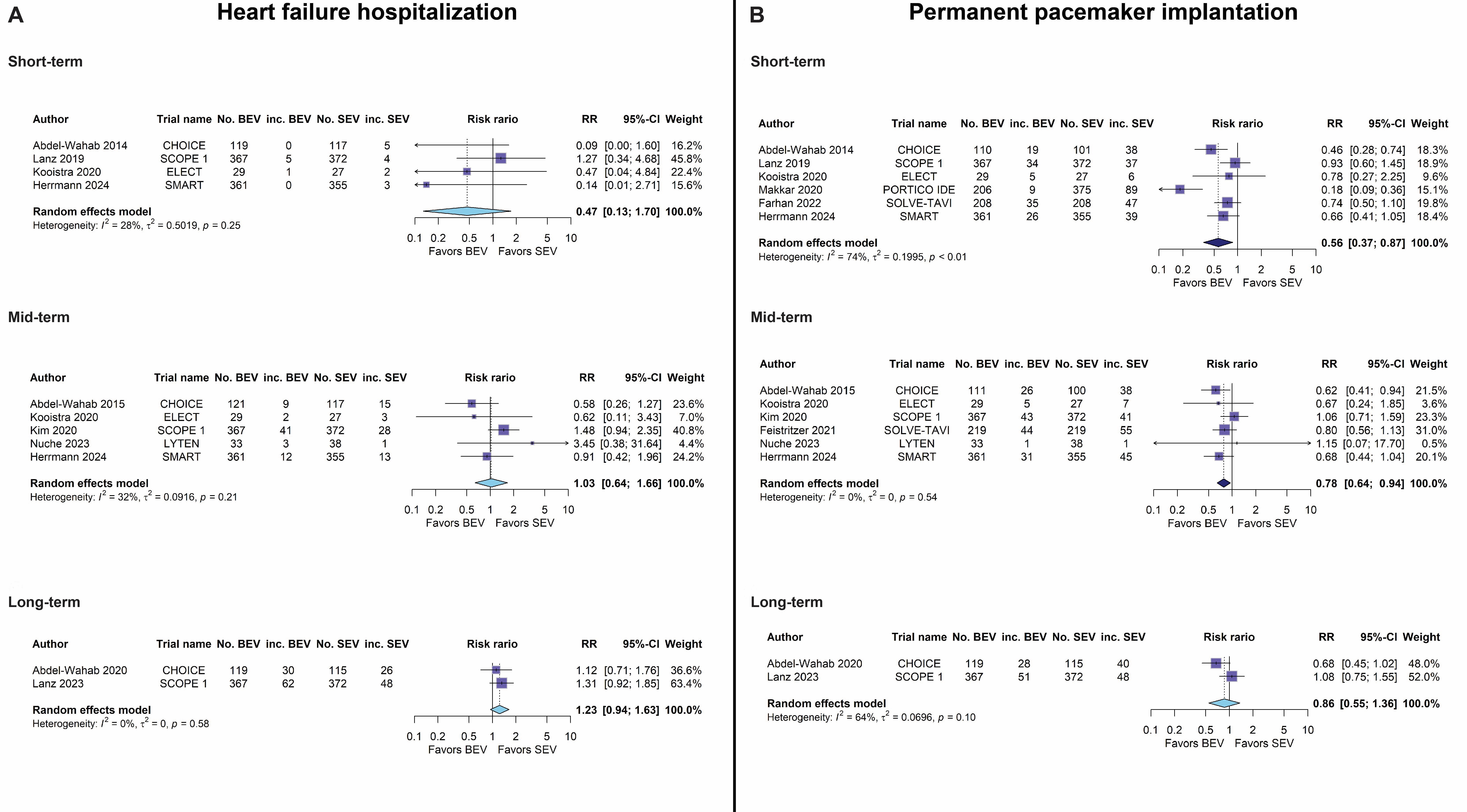

Supplement: Supplementary file 5 — Figure S5. Comparison of balloon‐expandable valves with self‐expanding valves for (A) heart failure hospitalization (B) and permanent pacemaker implantation at short‐term, midterm, and long‐term. BEV, balloon‐expandable valve; CI, confidence interval; RR, risk ratio; SEV, self‐expanding valve. [file CLC-48-e70134-s005.jpg]

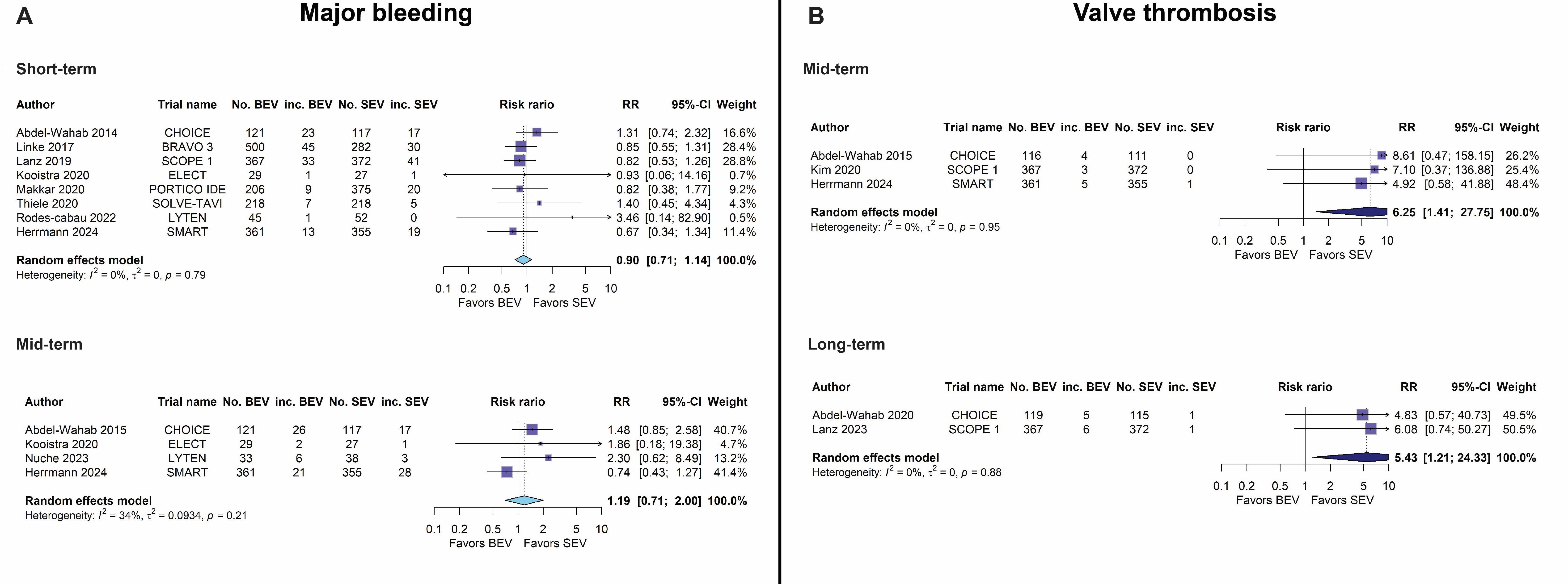

Supplement: Supplementary file 6 — Figure S6. Comparison of balloon‐expandable valves with self‐expanding valves for (A) major bleeding event (B) and clinical valve thrombosis at short‐term, midterm, and long‐term. BEV, balloon‐expandable valve; CI, confidence interval; RR, risk ratio; SEV, self‐expanding valve. [file CLC-48-e70134-s007.jpg]

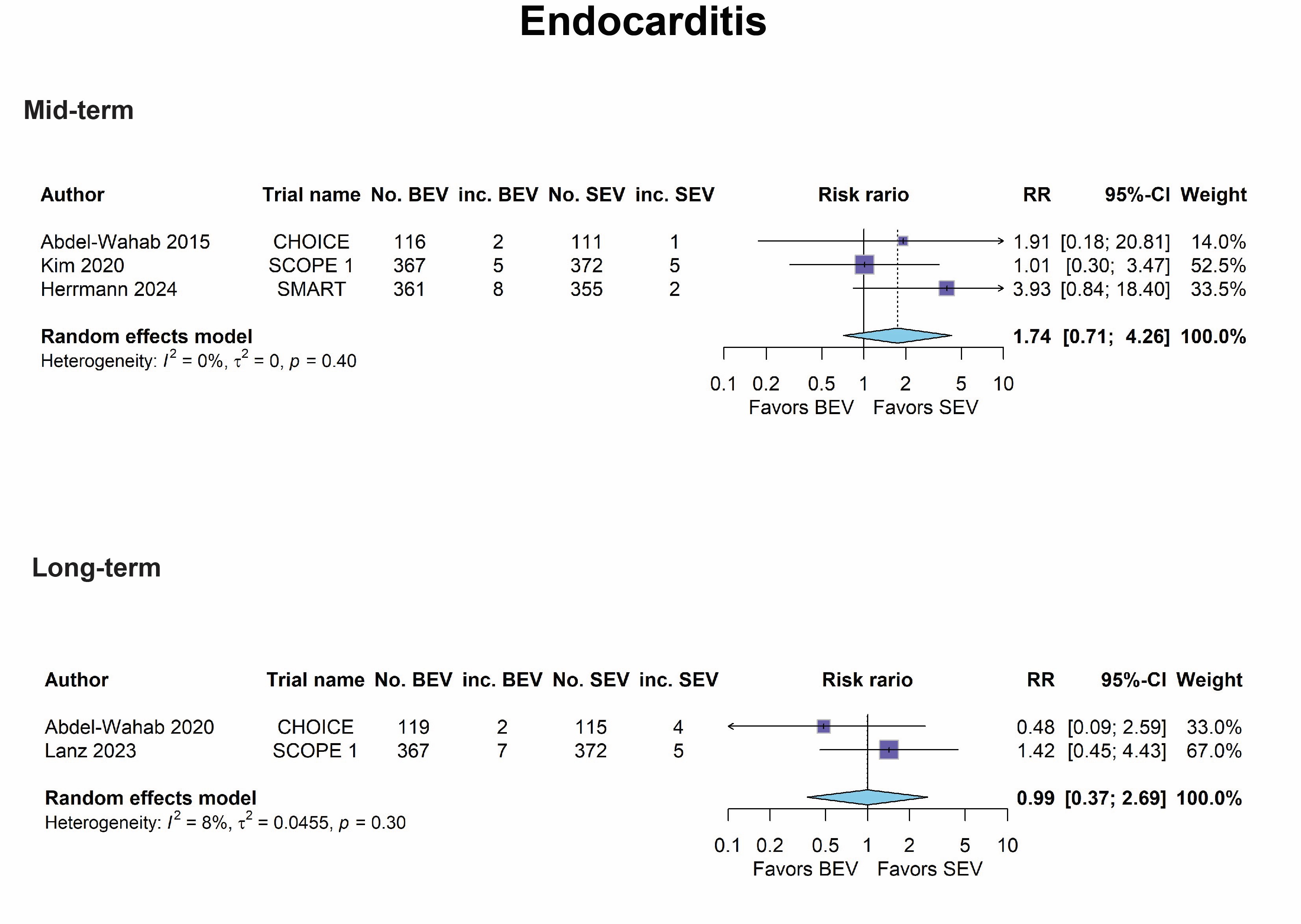

Supplement: Supplementary file 7 — Figure S7. Comparison of balloon‐expandable valves with self‐expanding valves for endocarditis at short‐term, and midterm. BEV, balloon‐expandable valve; CI, confidence interval; RR, risk ratio; SEV, self‐expanding valve. [file CLC-48-e70134-s006.jpg]

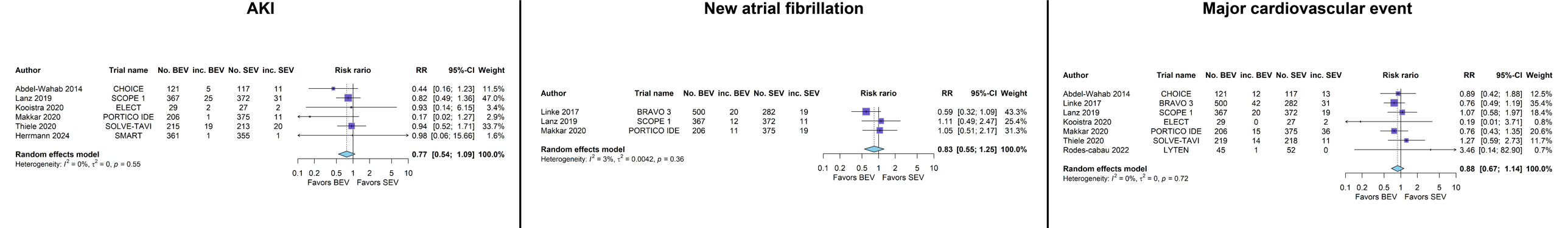

Supplement: Supplementary file 8 — Figure S8. Comparison of balloon‐expandable valves with self‐expanding valves for AKI, new atrial fibrillation, and major cardiovascular event at short‐term. AKI, acure kidney injury; BEV, balloon‐expandable valve; CI, confidence interval; RR, risk ratio; SEV, self‐expanding valve. [file CLC-48-e70134-s002.jpg]

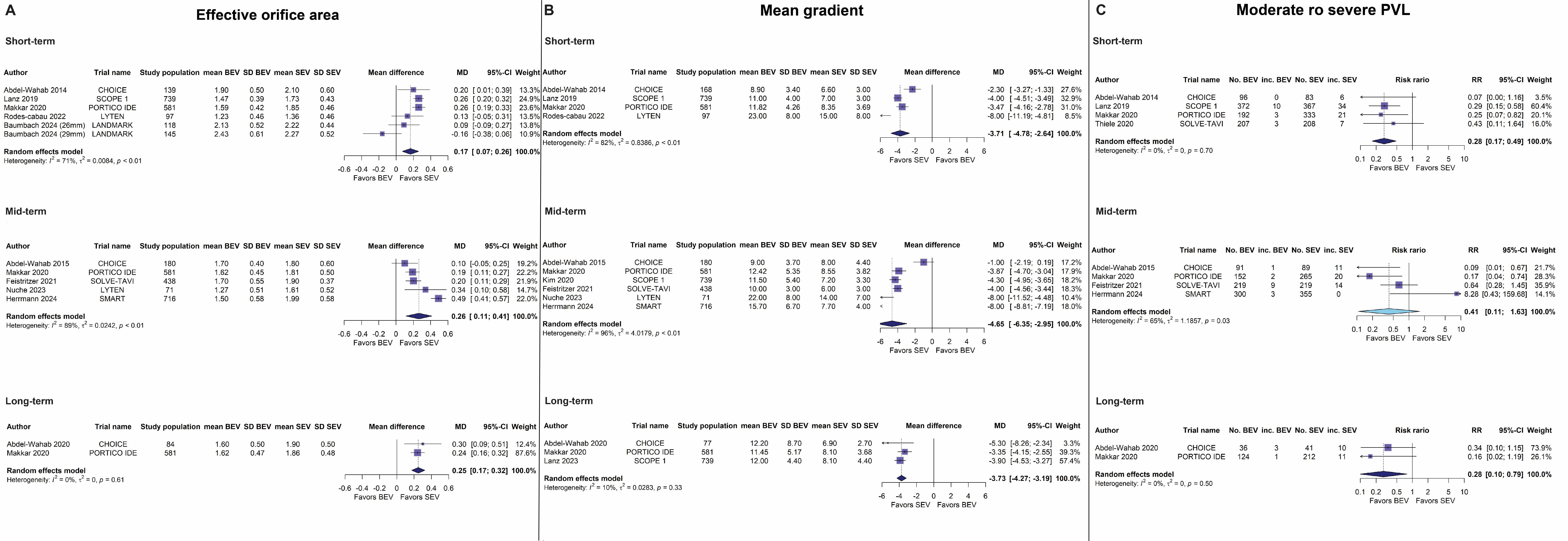

Supplement: Supplementary file 9 — Figure S9. Comparison of balloon‐expandable valves with self‐expanding valves for effective orifice area (cm2) (B) mean transvalvular pressure gradient (mmHg) and (C) moderate to severe PVL at short‐term, mid‐term, and long‐term. BEV, balloon‐expandable valve; CI, confidence interval; MD, mean difference; PVL, paravalvular leak; SEV, self‐expanding valve. [file CLC-48-e70134-s008.jpg]

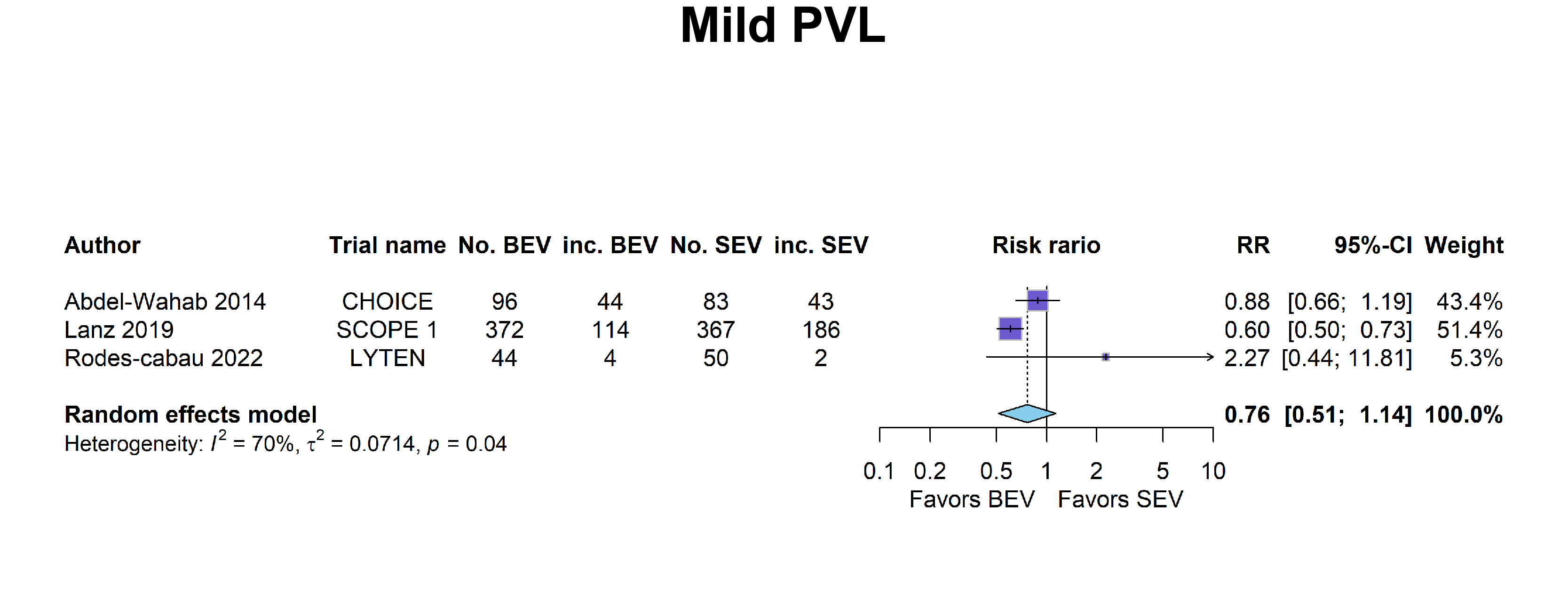

Supplement: Supplementary file 10 — Figure S10. Comparison of balloon‐expandable valves with self‐expanding valves for mild PVL at short‐term. BEV, balloon‐expandable valve; CI, confidence interval; PVL, paravalvular leak; RR, risk ratio; SEV, self‐expanding valve. [file CLC-48-e70134-s013.jpg]
