# Supplementary material for: Short‐Term, Mid‐Term, and Long‐Term Outcomes of Transcatheter Aortic Valve Replacement With Balloon‐Expandable Versus Self‐Expanding Valves: A Meta‐Analysis of Randomized Controlled Trials
Source: Clin Cardiol. 2025 Apr 19;48(4):e70134. doi: 10.1002/clc.70134 (PMC12008748; doi:10.1002/clc.70134)
Supplement: Supplementary file 11 — Supplementary Figures legends. [file CLC-48-e70134-s011.docx]

**Supplementary figure legends**

**Supplementary figure 1.** Summery bar plot for risk of bias assessment of the included studies.

**Supplementary figure 2.** Summery traffic light plot for risk of bias assessment of the included studies.

**Supplementary figure 3.** Comparison of balloon-expandable valves with self-expanding valves for (A) all-cause mortality (B) cardiovascular mortality and (C) stroke at short-term, mid-term, and long-term. BEV, balloon-expandable valve; CI, confidence interval; SEV, self-expanding valve; RR, Risk ratio.

**Supplementary figure 4.** Comparison of balloon-expandable valves with self-expanding valves for disabling and non-disabling stroke at short-term, mid-term, and long-term. BEV, balloon-expandable valve; CI, confidence interval; SEV, self-expanding valve; RR, Risk ratio.

**Supplementary figure 5.** Comparison of balloon-expandable valves with self-expanding valves for (A) heart failure hospitalization (B) and permanent pacemaker implantation at short-term, mid-term, and long-term. BEV, balloon-expandable valve; CI, confidence interval; SEV, self-expanding valve; RR, Risk ratio.

**Supplementary figure 6.** Comparison of balloon-expandable valves with self-expanding valves for (A) major bleeding event (B) and clinical valve thrombosis at short-term, mid-term, and long-term. BEV, balloon-expandable valve; CI, confidence interval; SEV, self-expanding valve; RR, Risk ratio.

**Supplementary figure 7.** Comparison of balloon-expandable valves with self-expanding valves for endocarditis at short-term, and mid-term. BEV, balloon-expandable valve; CI, confidence interval; SEV, self-expanding valve; RR, Risk ratio.

**Supplementary figure 8.** Comparison of balloon-expandable valves with self-expanding valves for AKI, new atrial fibrillation, and major cardiovascular event at short-term. AKI, acure kidney injury; BEV, balloon-expandable valve; CI, confidence interval; SEV, self-expanding valve; RR, Risk ratio.

**Supplementary figure 9.** Comparison of balloon-expandable valves with self-expanding valves for effective orifice area (cm2) (B) mean trans-valvular pressure gradient (mmHg) and (C) moderate to severe PVL at short-term, mid-term, and long-term. BEV, balloon-expandable valve; CI, confidence interval; PVL, para-valvular leak; SEV, self-expanding valve; MD, mean difference.

**Supplementary figure 10.** Comparison of balloon-expandable valves with self-expanding valves for mild PVL at short-term. BEV, balloon-expandable valve; CI, confidence interval; PVL, para-valvular leak; SEV, self-expanding valve; RR, Risk ratio.
