# Supplementary material for: Short‐Term, Mid‐Term, and Long‐Term Outcomes of Transcatheter Aortic Valve Replacement With Balloon‐Expandable Versus Self‐Expanding Valves: A Meta‐Analysis of Randomized Controlled Trials
Source: Clin Cardiol. 2025 Apr 19;48(4):e70134. doi: 10.1002/clc.70134 (PMC12008748; doi:10.1002/clc.70134)
Supplement: Supplementary file 12 — Supplementary Manuscript. [file CLC-48-e70134-s012.docx]

**Short-term, Mid-term, and Long-term Outcomes of Transcatheter Aortic Valve Replacement with Balloon-expandable Versus Self-expanding Valves:**

**A Meta-analysis of Randomized Controlled Trials**

**(Supplementary Manuscript)**

**Authors:**

Kiarash Tavakoli^1^, MD, MPH; Negin Sadat Hosseini Mohammadi^1^, MD, MPH; Pegah Bahiraie^2^, MD; Sahar Saeidi^1^, MD; Farhad Shaker^1^, MD, MPH; Arman Soltani Moghadam^1^, MD; Sara Montazeri Namin^1^, MD; Habib Rahban^3,4^, MD; Shubhadarshini Pawar^5^, MD, MBBS, MPH; Masih Tajdin^1,6^, MD; Hamidreza Soleimani^1,6^, MD; Yaser Jenab^1,6^, MD; Yousif Ahmad^7^, PHD; Fady Hany Iskander^8^, MD; Mohamad Alkhouli^9^, MD; Raj Makkar^5^, MD; Aakriti Gupta^5^, MD, MSC; Kaveh Hosseini^1,6^, MD, MPH.

**Affiliations:**

1. Tehran Heart Center, Cardiovascular Diseases Research Institute, Tehran University of Medical Sciences, Tehran, Iran.
2. School of Medicine, Shahid Beheshti University of Medical Sciences, Tehran, Iran.
3. Cardiovascular Research Foundation of Southern California, Beverly Hills, CA, USA.
4. Creighton University School of Medicine, St. Joseph Hospital and Medical Center, Department of Cardiovascular Disease, Phoenix, AZ, USA
5. Cedars Sinai Medical Center, Los Angeles, California, USA
6. Cardiac Primary Prevention Research Center, Cardiovascular Diseases Research Institute, Tehran University of Medical Sciences, Tehran, Iran.
7. Yale School of Medicine, Yale University, New Haven, CT, USA
8. Department of Cardiology, Medstar Union Memorial Hospital, 3333 North Calvert Street Johnston Professional Bldg Ste LL08, Baltimore, MD, 21218, USA
9. Department of Cardiology, Mayo Clinic School of Medicine, Rochester, MN, USA

**1.1 Subgroup Analysis of Clinical Outcomes**

Overall, the subgroup analyses based on the surgical risk of the patient, anatomical characteristics of the aortic annulus, SEV brand and generations did not reveal any statistically significant differences in the clinical outcomes between BEV and SEV across all follow-up intervals as presented in **Tables 1-4**.

**1.2 Subgroup Analysis of Hemodynamic Outcomes**

The larger EOA in SEV valves was more prominent in patients with high surgical risk through short-term (P-value: 0.009) and midterm (P-value: <0.0001) evaluations. On the other hand, the lower mean aortic valve gradient in SEV patients was more prominent in low surgical risk patients and newer generation SEV valves in short-term (P-value: 0.005, and 0.012, respectively) and mid-term (P-value <0.0001) endpoints. More specific comparisons demonstrated that lower mean aortic valve gradient in SEV patients was more prominent in Evolut valves. There was no significant difference between the subgroups based on the anatomical characteristics of the aortic annulus as presented in **Tables 1-4**.

**1.3 Procedural outcomes**

BEV procedures were associated with shorter procedure time (MD=7.58, 95% CI 4.51-10.64, P-value <0.0001), shorter fluoroscopic time (MD=1.79, 95% CI 0.05-3.54, P-value: 0.04) and lesser contrast volume (MD=21.61, 95% CI 17.02-26.19, P-value <0.0001) with low heterogeneity between the RCTs (I^2^:0%, Q: 0.47, P-value: 0.79; I^2^:30%, Q: 2.8, P-value: 0.24; and I^2^:29%, Q: 3.9, P-value: 0.07).

Moreover, BEV group reported lower unsuccessful procedure rate (RR=0.39, 95% CI 0.20-0.76, P-value: 0.006) and lower requirement for a second valve implantation (RR=0.15, 95% CI 0.08-0.30, P-value <0.0001). There was low heterogeneity for the need for second valve implantation (I^2^:0%, Q: 0.4, P-value: 0.99), but high heterogeneity for unsuccessful procedure rate (I^2^:63%, Q: 13, P-value: 0.01). According to the leave-one-out analysis, by excluding either Abdel-Wahab et al. (17) or Linke et al. (19) study, the device unsuccessful rate was no longer significantly different between the SEV and BEV cases (RR=0.47, 95% CI 0.22-1.04, and RR=0.48, 95% CI 0.23-1.02, respectively).

While the rate of balloon post-dilation was significantly lower in the BEV group (RR=0.62, 95% CI 0.48-0.79, P-value: 0.0002), pre-dilation was comparable between the SEV and BEV cases (RR=1.09, 95% CI 0.96-1.24, P-value: 0.19). The heterogeneity was low for post-dilation (I^2^:44%, Q: 19, P-value: 0.09) but high for pre-dilation (I^2^:91%, Q: 70, P-value <0.0001) as detailed in **Table 5**. The comparable rate of pre-dilation between SEV and BEV cases was consistent by excluding all studies in sensitivity analysis except for the Elnaggar et al. study (29), which resulted in a significantly higher rate of pre-dilation in the BEV group (RR=1.12, 95% CI 1.02-1.23).

| **Variable** | **Subgroup** | **Clinical outcome** | | | | | | | | | |
| --- | --- | --- | --- | --- | --- | --- | --- | --- | --- | --- | --- |
|  |  | **Short-term (30 days)** | | | | | **Mid-term (1 year)** | | | | |
|  |  | **No. studies** | **RR** | **95% CI** | **I^2^ (%)** | **P value of interaction** | **No. studies** | **RR** | **95% CI** | **I^2^ (%)** | **P value of interaction** |
| All-cause mortality | Not low risk | 6 | 0.51 | [0.31; 0.81] | 4.1% | 0.61 | 5 | 0.82 | [0.83; 1.93] | 0.0% | 0.054 |
|  | Low risk | 1 | 0.7 | [0.22; 2.12] | -- |  | 2 | 1.19 | [0.51; 1.05] | 36.0% |  |
| Cardiovascular mortality | Not low risk | 6 | 0.53 | [0.3; 0.91] | 8.6% | 0.662 | 5 | 0.83 | [0.5; 1.36] | 33.7% | 0.599 |
|  | Low risk | 1 | 0.70 | [0.22; 2.19] | -- |  | 1 | 1.06 | [0.49; 2.3] | -- |  |
| Stroke | Not low risk | 6 | 1.32 | [0.69; 2.54] | 40.6% | 0.281 | 5 | 1.49 | [0.66; 3.34] | 63.0% | 0.23 |
|  | Low risk | 1 | 0.73 | [0.31; 1.72] | -- |  | 2 | 0.79 | [0.41; 1.52] | 0.0% |  |
| Major bleeding | Not low risk | 6 | 0.92 | [0.72; 1.18] | 0.0% | 0.5 | 4 | 0.80 | [0.62; 1.02] | 15.4% | 0.537 |
|  | Low risk | 2 | 0.72 | [0.36; 1.42] | 0.0% |  | 2 | 0.68 | [0.4473; 1.05] | 0.0% |  |
| Heart failure hospitalization | Not low risk | 3 | 0.56 | [0.13; 2.45] | 35.0% | 0.409 | 3 | 0.93 | [0.44; 1.9496] | 56.4% | 0.730 |
|  | Low risk | 1 | 0.14 | [0.007; 2.7] | -- |  | 2 | 1.16 | [0.41; 3.24] | 20.2% |  |
| Clinical valve thrombosis | Not low risk | NA | NA | NA | NA | NA | 2 | 7.83 | [0.98; 62.37] | 0.0% | 0.759 |
|  | Low risk | NA | NA | NA | NA |  | 1 | 4.91 | [0.57; 41.87] | -- |  |
| Endocarditis | Not low risk | NA | NA | NA | NA | NA | 2 | 1.15 | [0.38; 3.46] | 0.0% | 0.205 |
|  | Low risk | NA | NA | NA | NA |  | 1 | 3.93 | [0.84; 18.39] | -- |  |
| Permanent pace maker | Not low risk | 5 | 0.54 | [0.31; 0.93] | 79.4% | 0.606 | NA | NA | NA | NA | NA |
|  | Low risk | 1 | 0.65 | [0.4; 1.05] | -- |  | NA | NA | NA | NA |  |
| Major cardiovascular event | Not low risk | 6 | 0.86 | [0.66; 1.13] | 0.0% | 0.395 | NA | NA | NA | NA | NA |
|  | Low risk | 1 | 3.46 | [0.14; 82.89] | -- |  | NA | NA | NA | NA |  |
| AKI | Not low risk | 5 | 0.76 | [0.53; 1.08] | 0.0% | 0.859 | NA | NA | NA | NA | NA |
|  | Low risk | 1 | 0.98 | [0.06; 15.6] | -- |  | NA | NA | NA | NA |  |
| **Variable** | **Subgroup** | **Hemodynamic outcome** | | | | | | | | | |
|  |  | **No. studies** | **MD** | **95% CI** | **I^2^ (%)** | **P value of interaction** | **No. studies** | **MD** | **95% CI** | **I^2^ (%)** | **P value of interaction** |
| Mean gradient | Not low risk | 3 | -3.33 | [-4.21; -2.45] | 78.8% | **0.0058*** | 4 | -3.4 | [-4.47; -2.32] | 87.6% | **<0.0001*** |
|  | Low risk | 1 | -8 | [-11.19; -4.8] | -- |  | 2 | -8 | [-8.78; -7.21] | 0.0% |  |
| Effective orifice area | Not low risk | 3 | 0.25 | [0.21; 0.3] | 53.9% | **0.0098*** | 2 | 0.45 | [0.33; 0.57] | 25.0% | **<0.0001*** |
|  | Low risk | 3 | 0.03 | [-0.13; 0.19] | 0.0% |  | 3 | 0.18 | [0.12; 0.23] | 0.0% |  |
| Moderate to severe PVL | Not low risk | NA | NA | NA | NA | NA | 3 | 0.26 | [0.07; 0.94] | 60.6% | **0.036*** |
|  | Low risk | NA | NA | NA | NA |  | 1 | 8.28 | [0.42; 159.68] | -- |  |

**Table 1.** Subgroup analysis of clinical and hemodynamic outcomes based on surgical risk.

AKI, Acute kidney injury; CI, Confidence interval; PVL, paravalvular leak; RR, risk ratio.

| **Variable** | **Subgroup** | **Clinical outcome** | | | | | | | | | |
| --- | --- | --- | --- | --- | --- | --- | --- | --- | --- | --- | --- |
|  |  | **Short-term (30 days)** | | | | | **Mid-term (1 year)** | | | | |
|  |  | **No. studies** | **RR** | **95% CI** | **I^2^ (%)** | **P value of interaction** | **No. studies** | **RR** | **95% CI** | **I^2^ (%)** | **P value of interaction** |
| All-cause mortality | Old valve | 3 | 0.55 | [0.32; 0.94] | 0.0% | 0.737 | 2 | 0.47 | [0.03; 7.41] | 73.6% | 0.686 |
|  | New valve | 4 | 0.47 | [0.20; 1.06] | 28.5% |  | 5 | 0.83 | [0.64; 1.08] | 7.3% |  |
| Cardiovascular mortality | Old valve | 3 | 0.57 | [0.32; 1] | 0.0% | 0.813 | 2 | 0.46 | [0.03; 7.15] | 72.8% | 0.6944 |
|  | New valve | 4 | 0.5 | [0.21; 1.22] | 25.2% |  | 4 | 0.80 | [0.55; 1.16] | 0.0% |  |
| Stroke | Old valve | 3 | 0.96 | [0.33; 2.77] | 42.3% | 0.626 | 2 | 0.60 | [0.01; 20.11] | 81.4% | 0.7 |
|  | New valve | 4 | 1.33 | [0.62; 2.88] | 50.4% |  | 5 | 1.20 | [0.66; 2.16] | 47.1% |  |
| MACE | Old valve | 3 | 0.69 | [0.20; 2.31] | 66.5% | 0.673 | NA | NA | NA | NA | NA |
|  | New valve | 1 | 0.90 | [0.61; 1.23] | -- |  | NA | NA | NA | NA |  |
| Major bleeding | Old valve | 3 | 0.99 | [0.7; 1.4] | 0.0% | 0.453 | 2 | 1.49 | [0.87; 2.57] | 0.0% | 0.624 |
|  | New valve | 5 | 0.82 | [0.6; 1.13] | 0.0% |  | 2 | 1.11 | [0.38; 3.23] | 59.8% |  |
| Heart failure hospitalization | Old valve | 2 | 0.24 | [0.04; 1.48] | 0.0% | 0.495 | 2 | 0.58 | [0.28; 1.19] | 0.0% | **0.04*** |
|  | New valve | 2 | 0.62 | [0.08; 4.92] | 46.4% |  | 3 | 1.34 | [0.91; 1.98] | 0.0% |  |
| Clinical valve thrombosis | Old valve | NA | NA | NA | NA | NA | 1 | 8.61 | [0.46; 158.14] | -- | 0.801 |
|  | New valve | NA | NA | NA | NA |  | 2 | 5.57 | [0.98; 31.62] | 0.0% |  |
| Endocarditis | Old valve | NA | NA | NA | NA | NA | 1 | 1.91 | [0.17; 20.80] | -- | 0.977 |
|  | New valve | NA | NA | NA | NA |  | 2 | 1.84 | [0.48; 6.94] | 45.6% |  |
| Permanent pace maker | Old valve | 2 | 0.5 | [0.32; 0.77] | 0.0% | 0.758 | 2 | 0.62 | [0.42; 0.91] | 0.0% | 0.195 |
|  | New valve | 4 | 0.56 | [0.31; 1.01] | 83.2% |  | 4 | 0.83 | [0.66; 1.04] | 0.0% |  |
| Major cardiovascular event | Old valve | 3 | 0.77 | [0.53; 1.13] | 0.0% | 0.38 | NA | NA | NA | NA | NA |
|  | New valve | 4 | 0.98 | [0.68; 1.41] | 0.0% |  | NA | NA | NA | NA |  |
| AKI | Old valve | 2 | 0.52 | [0.21; 1.28] | 0.0% | 0.362 | NA | NA | NA | NA | NA |
|  | New valve | 4 | 0.82 | [0.56; 1.19] | 0.0% |  | NA | NA | NA | NA |  |
| **Variable** | **Subgroup** | **Hemodynamic outcome** | | | | | | | | | |
|  |  | **No. studies** | **MD** | **95% CI** | **I^2^ (%)** | **P value of interaction** | **No. studies** | **MD** | **95% CI** | **I^2^ (%)** | **P value of interaction** |
| Mean gradient | Old valve | 1 | -2.30 | [-3.26; -1.33] | -- | **0.012*** | 1 | -1.00 | [-2.18; 0.19] | -- | **<0.0001*** |
|  | New valve | 3 | -4.13 | [-5.18; -3.07] | 74.9% |  | 5 | -5.36 | [-7.02; -3.71] | 94.9% |  |
| Effective orifice area | Old valve | 1 | 0.2 | [0.01; 0.38] | -- | 0.684 | 1 | 0.1 | [-0.04; 0.24] | -- | 0.076 |
|  | New valve | 5 | 0.15 | [0.04; 0.26] | 76.4% |  | 4 | 0.3 | [ 0.13; 0.47] | 90.5% |  |
| Moderate to severe PVL | Old valve | 1 | 0.06 | [0.004; 1.16] | -- | 0.311 | 1 | 0.08 | [0.01; 0.67] | -- | 0.1292 |
|  | New valve | 3 | 0.29 | [0.17; 0.51] | 0.0% |  | 3 | 0.62 | [0.13; 2.83] | 65.7% |  |

**Table 2.** Subgroup analysis of clinical and hemodynamic outcomes based on valve generation*.*

AKI, Acute kidney injury; CI, Confidence interval; PVL, paravalvular leak; RR, risk ratio.

| **Variable** | **Subgroup** | **Clinical outcome** | | | | | | | | | | |
| --- | --- | --- | --- | --- | --- | --- | --- | --- | --- | --- | --- | --- |
|  |  | **Short-term (30 days)** | | | | | **Mid-term (1 year)** | | | | | |
|  |  | **No. studies** | **RR** | **95% CI** | **I^2^ (%)** | **P value of interaction** | **No. studies** | **RR** | **95% CI** | **I^2^ (%)** | **P value of interaction** |  |
| All-cause mortality | Non- small annulus | 5 | 0.45 | [0.25; 0.80] | 12.1% | 0.321 | 4 | 0.73 | [0.51; 1.05] | 36.0% | **0.054*** |  |
|  | small annulus | 2 | 0.75 | [0.33; 1.69] | 0.0% |  | 3 | 1.26 | [0.83; 1.93] | 0.0% |  |  |
| Cardiovascular mortality | Non- small annulus | 5 | 0.46 | [0.25; 0.85] | 8.7% | 0.282 | 4 | 0.71 | [0.41; 1.21] | 23.6% | 0.178 |  |
|  | small annulus | 2 | 0.81 | [0.35; 1.87] | 0.0% |  | 2 | 1.19 | [0.69; 2.02] | 0.0% |  |  |
| Stroke | Non- small annulus | 5 | 1.21 | [0.57; 2.57] | 47.1% | 0.928 | 4 | 1.27 | [0.48; 3.36] | 67.4% | 0.921 |  |
|  | small annulus | 2 | 1.14 | [0.39; 3.33] | 48.2% |  | 3 | 1.19 | [0.46; 3.07] | 44.1% |  |  |
| MACE | Non- small annulus | 3 | 0.71 | [0.41; 1.21] | 58.8% | 0.127 | NA | NA | NA | NA | NA |  |
|  | small annulus | 1 | 1.93 | [0.59; 6.24] | -- |  | NA | NA | NA | NA |  |  |
| Major bleeding | Non- small annulus | 5 | 0.85 | [0.64; 1.12] | 0.0% | 0.602 | 1 | 1.86 | [0.17; 19.37] | -- | 0.714 |  |
|  | small annulus | 3 | 1.01 | [0.57; 1.78] | 25.8% |  | 3 | 1.18 | [0.64; 2.17] | 54.3% |  |  |
| Heart failure hospitalization | Non- small annulus | 2 | 0.99 | [0.31; 3.12] | 0.0% | 0.068 | 2 | 1.4 | [0.89; 2.17] | 0.0% | 0.166 |  |
|  | small annulus | 2 | 0.11 | [0.01; 0.87] | 0.0% |  | 3 | 0.81 | [0.44; 1.51] | 17.7% |  |  |
| Valve thrombosis | Non- small annulus | NA | NA | NA | NA | NA | 1 | 7.09 | [0.36; 136.88] | -- | 0.922 |  |
|  | small annulus | NA | NA | NA | NA |  | 2 | 5.98 | [1.06; 33.61] | 0.0% |  |  |
| Permanent pace maker | Non- small annulus | 4 | 0.56 | [0.27; 1.14] | 83.6% | 0.94 | 3 | 0.88 | [0.68; 1.14] | 0.0% | 0.121 |  |
|  | small annulus | 2 | 0.54 | [0.38; 0.78] | 7.2% |  | 3 | 0.64 | [0.48; 0.87] | 0.0% |  |  |
| Major cardiovascular event | Non- small annulus | 5 | 0.86 | [0.65; 1.14] | 0.0% | 0.798 | NA | NA | NA | NA | NA |  |
|  | small annulus | 2 | 0.95 | [0.46; 1.97] | 0.0% |  | NA | NA | NA | NA |  |  |
| AKI | Non- small annulus | 4 | 0.82 | [0.56; 1.19] | 0.0% | 0.314 | NA | NA | NA | NA | NA |  |
|  | small annulus | 2 | 0.48 | [0.18; 1.26] | 0.0% |  | NA | NA | NA | NA |  |  |
| **Variable** | **Subgroup** | **Hemodynamic outcome** | | | | | | | | | | |
|  |  | **No. studies** | **MD** | **95% CI** | **I^2^ (%)** | **P value of interaction** | **No. studies** | **MD** | **95% CI** | **I^2^ (%)** | **P value of interaction** |  |
| Mean gradient | Non- small annulus | 2 | -3.78 | [ -4.29; -3.28] | 31.7% | 0.68 | 3 | -4.07 | [ -4.45; -3.69] | 0.0% | 0.585 |  |
|  | small annulus | 2 | -4.93 | [-10.50; 0.63] | 91.1% |  | 3 | -5.58 | [-10.98; -0.17] | 97.8% |  |  |
| Effective orifice area | Non- small annulus | 2 | 0.26 | [0.21; 0.3] | 0.0% | **0.0137*** | 2 | 0.19 | [0.13; 0.25] | 0.0% | 0.4 |  |
|  | small annulus | 4 | 0.07 | [-0.06; 0.21] | 53.0% |  | 3 | 0.31 | [0.04; 0.58] | 90.0% |  |  |
| Moderate to severe PVL | Non- small annulus | 1 | 0.06 | [0.003; 1.16] | 0.0% | 0.311 | 2 | 0.38 | [0.10; 1.39] | 60.8% | 0.774 |  |
|  | small annulus | 3 | 0.29 | [0.17; 0.51] | -- |  | 2 | 0.75 | [0.01; 63.63] | 83.7% |  |  |

**Table 3.** Subgroup analysis of clinical and hemodynamic outcomes based on the anatomical characteristics of the aortic annulus*.*

AKI, Acute kidney injury; CI, Confidence interval; PVL, paravalvular leak; RR, risk ratio.

| **Variable** | **Subgroup** | **Clinical outcome** | | | | | | | | | |
| --- | --- | --- | --- | --- | --- | --- | --- | --- | --- | --- | --- |
|  |  | **Short-term (30 days)** | | | | | **Mid-term (1 year)** | | | | |
|  |  | **No. studies** | **RR** | **95% CI** | **I^2^ (%)** | **P value of interaction** | **No. studies** | **RR** | **95% CI** | **I^2^ (%)** | **P value of interaction** |
| All-cause mortality | Medtronic CoreValve | 3 | 0.55 | [0.32; 0.94] | 0.0% | 0.306 | 2 | 0.47 | [0.03; 7.41] | 73.6% | 0.263 |
|  | Medtronic Evolut | 2 | 0.71 | [0.32; 1.57] | 0.0% |  | 3 | 1.05 | [0.74; 1.49] | 0.0% |  |
|  | Portico | 1 | 0.05 | [0.01; 0.86] | -- |  | 1 | 0.57 | [0.34; 0.96] | -- |  |
|  | ACURATE | 1 | 0.34 | [0.09; 1.23] | -- |  | 1 | 0.76 | [0.48; 1.19] | -- |  |
| Cardiovascular mortality | Medtronic CoreValve | 3 | 0.57 | [0.32; 1] | 0.0% | 0.340 | 2 | 0.46 | [0.03; 7.15] | 72.8% | 0.559 |
|  | Medtronic Evolut | 2 | 0.79 | [0.31; 1.99] | 0.0% |  | 2 | 1.19 | [0.59; 2.39] | 0.0% |  |
|  | Portico | 1 | 0.05 | [0.03; 0.97] | -- |  | 1 | 0.61 | [0.31; 1.17] | -- |  |
|  | ACURATE | 1 | 0.38 | [0.10; 1.42] | -- |  | 1 | 0.77 | [0.43; 1.37] | -- |  |
| Stroke | Medtronic CoreValve | 3 | 0.96 | [0.33; 2.77] | 42.3% | 0.828 | 2 | 0.60 | [0.01; 20.11] | 81.4% | 0.814 |
|  | Medtronic Evolut | 2 | 2.29 | [0.16; 32] | 82.6% |  | 3 | 1.50 | [0.29; 7.84] | 71.5 |  |
|  | Portico | 1 | 0.99 | [0.37; 2.65] | -- |  | 1 | 1.36 | [0.66; 2.83] | -- |  |
|  | ACURATE | 1 | 1.59 | [0.62; 4.06] | -- |  | 1 | 0.89 | [0.45; 1.76] | -- |  |
| MACE | Medtronic CoreValve | 3 | 0.69 | [0.20; 2.31] | 66.5% | 0.673 | NA | NA | NA | NA | NA |
|  | Medtronic Evolut | 1 | 0.90 | [0.61; 1.23] | -- |  | NA | NA | NA | NA |  |
| Major bleeding | Medtronic CoreValve | 3 | 0.99 | [0.7; 1.4] | 0.0% | 0.899 | 2 | 1.49 | [0.87; 2.57] | 0.0% | 0.624 |
|  | Medtronic Evolut | 3 | 0.86 | [0.48; 1.54] | 0.0% |  | 2 | 1.11 | [0.38; 3.23] | 59.8% |  |
|  | Portico | 1 | 0.82 | [0.38; 1.76] | -- |  | NA | NA | NA | NA |  |
|  | ACURATE | 1 | 0.81 | [0.53; 1.26] | -- |  | NA | NA | NA | NA |  |
| Heart failure hospitalization | Medtronic CoreValve | 2 | 0.24 | [0.04; 1.48] | 0.0% | 0.207 | 2 | 0.58 | [0.28; 1.19] | 0.0% | 0.1 |
|  | Medtronic Evolut | 1 | 0.14 | [0.01; 2.70] | -- |  | 2 | 1.16 | [0.42; 3.24] | 20.2% |  |
|  | ACURATE | 1 | 1.27 | [0.34; 4.68] | -- |  | 1 | 1.48 | [0.93; 2.34] | -- |  |
| Clinical valve thrombosis | Medtronic CoreValve | NA | NA | NA | NA | NA | 1 | 8.61 | [0.46; 158.14] | -- | 0.950 |
|  | Medtronic Evolut | NA | NA | NA | NA |  | 1 | 4.91 | [0.57; 41.87] | -- |  |
|  | ACURATE | NA | NA | NA | NA |  | 1 | 7.09 | [0.37; 136.88] | -- |  |
| Endocarditis | Medtronic CoreValve | NA | NA | NA | NA | NA | 1 | 1.91 | [0.17; 20.80] | -- | 0.402 |
|  | Medtronic Evolut | NA | NA | NA | NA |  | 1 | 3.93 | [0.84; 18.39] | -- |  |
|  | ACURATE | NA | NA | NA | NA |  | 1 | 1.01 | [0.29; 3.47] | -- |  |
| Permanent pace maker | Medtronic CoreValve | 2 | 0.5 | [0.32; 0.77] | 0.0% | **0.0005*** | 2 | 0.62 | [0.42; 0.91] | 0.0% | 0.163 |
|  | Medtronic Evolut | 2 | 0.7 | [0.52; 0.95] | 0.0% |  | 3 | 0.75 | [0.57; 0.98] | 0.0% |  |
|  | Portico | 1 | 0.18 | [0.09; 0.35] | -- |  | NA | NA | NA | NA |  |
|  | ACURATE | 1 | 0.93 | [0.59; 1.45] | -- |  | 1 | 1.06 | [0.71; 1.59] | -- |  |
| Major cardiovascular event | Medtronic CoreValve | 3 | 0.77 | [0.53; 1.13] | 0.0% | 0.518 | NA | NA | NA | NA | NA |
|  | Medtronic Evolut | 2 | 1.34 | [0.63; 2.82] | 0.0% |  | NA | NA | NA | NA |  |
|  | Portico | 1 | 0.76 | [0.43; 1.35] | -- |  | NA | NA | NA | NA |  |
|  | ACURATE | 1 | 1.06 | [0.58; 1.96] | -- |  | NA | NA | NA | NA |  |
| AKI | Medtronic CoreValve | 2 | 0.52 | [0.21; 1.28] | 0.0% | 0.332 | NA | NA | NA | NA | NA |
|  | Medtronic Evolut | 2 | 0.94 | [0.52; 1.69] | 0.0% |  | NA | NA | NA | NA |  |
|  | Portico | 1 | 0.17 | [0.02; 1.27] | -- |  | NA | NA | NA | NA |  |
|  | ACURATE | 1 | 0.82 | [0.49; 1.36] | -- |  | NA | NA | NA | NA |  |
| **Variable** | **Subgroup** | **Hemodynamic outcome** | | | | | | | | | |
|  |  | **No. studies** | **MD** | **95% CI** | **I^2^ (%)** | **P value of interaction** | **No. studies** | **MD** | **95% CI** | **I^2^ (%)** | **P value of interaction** |
| Mean gradient | Medtronic CoreValve | 1 | -2.30 | [-3.26; -1.33] | -- | **0.0008*** | 1 | -1.00 | [-2.18; 0.19] | -- | **<0.0001*** |
|  | Medtronic Evolut | 1 | -8.00 | [-11.19; -4.80] | -- |  | 3 | -6.51 | [-9.82; -3.21] | 97.0% |  |
|  | Portico | 1 | -3.47 | [-4.16; -2.78] | -- |  | 1 | -3.87 | [-4.69; -3.04] | -- |  |
|  | ACURATE | 1 | -4.00 | [-4.51; -3.49] | -- |  | 1 | -4.30 | [-4.95; -3.65] | -- |  |
| Effective orifice area | Medtronic CoreValve | 1 | 0.2 | [0.01; 0.38] | -- | 0.070 | 1 | 0.1 | [-0.04; 0.24] | -- | 0.195 |
|  | Medtronic Evolut | 3 | 0.03 | [-0.13; 0.19] | 53.9% |  | 3 | 0.34 | [0.12; 0.56] | 90.8% |  |
|  | Portico | 1 | 0.26 | [0.18; 0.33] | -- |  | 1 | 0.19 | [0.11; 0.26] | -- |  |
|  | ACURATE | 1 | 0.26 | [0.20; 0.32] | -- |  | NA | NA | NA | NA |  |
| Moderate to severe PVL | Medtronic CoreValve | 1 | 0.06 | [0.004; 1.16] | -- | 0.702 | 1 | 0.08 | [0.01; 0.67] | -- | 0.187 |
|  | Medtronic Evolut | 1 | 0.43 | [0.11; 1.64] | -- |  | 2 | 1.55 | [0.14; 17.76] | 64.1% |  |
|  | Portico | 1 | 0.24 | [0.07; 0.82] | -- |  | 1 | 0.17 | [0.04; 0.73] | -- |  |
|  | ACURATE | 1 | 0.29 | [0.14; 0.58] | -- |  | NA | NA | NA | NA |  |

**Table 2.** Subgroup analysis of clinical and hemodynamic outcomes based on self-expanding valve brands*.*

AKI, Acute kidney injury; CI, Confidence interval; PVL, paravalvular leak; RR, risk ratio.

| **Variable** | **No. studies** | **RR/MD** | **95% CI** | **P value** | **Tau^2^** | **I^2^ (%)** |
| --- | --- | --- | --- | --- | --- | --- |
| Procedure duration (minute) | 3 | 7.58 | [4.51 – 10.64] | **< 0.0001*** | 0 | 0.0 |
| Contrast volume (ml) | 6 | 21.61 | [17.02 – 26.19] | **< 0.0001*** | 8.97 | 29.1 |
| Fluoroscopic duration (minute) | 3 | 1.79 | [0.05 – 3.54] | **0.043*** | 0.91 | 29.6 |
| Device unsuccessful | 7 | 0.39 | [0.2 – 0.76] | **0.006*** | 0.39 | 63.5 |
| Second valve requirement | 7 | 0.15 | [0.08 – 0.3] | **< 0.0001*** | 0 | 0.0 |
| Pre-dilation | 7 | 1.09 | [0.96 – 1.24] | 0.193 | 0.01 | 91.5 |
| Post-dilation | 7 | 0.62 | [0.48 – 0.79] | **0.0002*** | 0.04 | 44 |

**Table 5.** Procedural outcome.

CI, Confidence interval; MD, Mean difference; RR, Risk ratio.
