# Supplementary material for: Short‐Term, Mid‐Term, and Long‐Term Outcomes of Transcatheter Aortic Valve Replacement With Balloon‐Expandable Versus Self‐Expanding Valves: A Meta‐Analysis of Randomized Controlled Trials
Source: Clin Cardiol. 2025 Apr 19;48(4):e70134. doi: 10.1002/clc.70134 (PMC12008748; doi:10.1002/clc.70134)
Supplement: Supplementary file 13 — Supplementary Tables. [file CLC-48-e70134-s001.docx]

| **Section and Topic** | **Item #** | **Checklist item** | **Location where item is reported** |
| --- | --- | --- | --- |
| **TITLE** | | |  |
| Title | 1 | Identify the report as a systematic review. | Reported on title page. |
| **ABSTRACT** | | |  |
| Abstract | 2 | See the PRISMA 2020 for Abstracts checklist. | Abstract provided by PRISMA 2020 abstracts checklist and the Journal’s instructions to authors. |
| **INTRODUCTION** | | |  |
| Rationale | 3 | Describe the rationale for the review in the context of existing knowledge. | Rationale for conducting this study is provided in the second paragraph of ‘Introduction’. |
| Objectives | 4 | Provide an explicit statement of the objective(s) or question(s) the review addresses. | Explicitly stated in last paragraph of ‘Introduction’. |
| **METHODS** | | |  |
| Eligibility criteria | 5 | Specify the inclusion and exclusion criteria for the review and how studies were grouped for the syntheses. | Explained under ‘Study selection and eligibility criteria’. |
| Information sources | 6 | Specify all databases, registers, websites, organisations, reference lists and other sources searched or consulted to identify studies. Specify the date when each source was last searched or consulted. | Reported under ‘Literature search strategy’. |
| Search strategy | 7 | Present the full search strategies for all databases, registers and websites, including any filters and limits used. | Provided in Supplementary Table 2. |
| Selection process | 8 | Specify the methods used to decide whether a study met the inclusion criteria of the review, including how many reviewers screened each record and each report retrieved, whether they worked independently, and if applicable, details of automation tools used in the process. | Explained under ‘Study selection and eligibility criteria’. |
| Data collection process | 9 | Specify the methods used to collect data from reports, including how many reviewers collected data from each report, whether they worked independently, any processes for obtaining or confirming data from study investigators, and if applicable, details of automation tools used in the process. | Reported under ‘Data extraction’. |
| Data items | 10a | List and define all outcomes for which data were sought. Specify whether all results that were compatible with each outcome domain in each study were sought (e.g. for all measures, time points, analyses), and if not, the methods used to decide which results to collect. | Reported under ‘Data extraction’, and provided in Table 1 and Supplementary Table 4. |
|  | 10b | List and define all other variables for which data were sought (e.g. participant and intervention characteristics, funding sources). Describe any assumptions made about any missing or unclear information. | Reported under ‘Data extraction’ and Supplementary Table 3. |
| Study risk of bias assessment | 11 | Specify the methods used to assess risk of bias in the included studies, including details of the tool(s) used, how many reviewers assessed each study and whether they worked independently, and if applicable, details of automation tools used in the process. | Reported under ‘Risk of bias and quality assessment’. |
| Effect measures | 12 | Specify for each outcome the effect measure(s) (e.g. risk ratio, mean difference) used in the synthesis or presentation of results. | Reported under ‘Statistical analyses’. |
| Synthesis methods | 13a | Describe the processes used to decide which studies were eligible for each synthesis (e.g. tabulating the study intervention characteristics and comparing against the planned groups for each synthesis (item #5)). | Explained under Study selection and eligibility criteria’ and ‘Statistical analyses’, respectively. |
|  | 13b | Describe any methods required to prepare the data for presentation or synthesis, such as handling of missing summary statistics, or data conversions. | Reported under ‘Statistical analyses’. |
|  | 13c | Describe any methods used to tabulate or visually display results of individual studies and syntheses. | Reported under ‘Statistical analyses’. |
|  | 13d | Describe any methods used to synthesize results and provide a rationale for the choice(s). If meta-analysis was performed, describe the model(s), method(s) to identify the presence and extent of statistical heterogeneity, and software package(s) used. | Reported under ‘Statistical analyses’. |
|  | 13e | Describe any methods used to explore possible causes of heterogeneity among study results (e.g. subgroup analysis, meta-regression). | Reported under ‘Statistical analyses’. |
|  | 13f | Describe any sensitivity analyses conducted to assess robustness of the synthesized results. | Reported under ‘Statistical analyses’. |
| Reporting bias assessment | 14 | Describe any methods used to assess risk of bias due to missing results in a synthesis (arising from reporting biases). | No publication bias analyses were conducted due to the limited number of included studies, however, our explanation was provided under ‘Limitations’. |
| Certainty assessment | 15 | Describe any methods used to assess certainty (or confidence) in the body of evidence for an outcome. | Reported under ‘Risk of bias and quality assessment’. |
| **RESULTS** | | |  |
| Study selection | 16a | Describe the results of the search and selection process, from the number of records identified in the search to the number of studies included in the review, ideally using a flow diagram. | Explained under first paragraph of ‘Results’ and the flow diagram depicted in Figure 1. |
|  | 16b | Cite studies that might appear to meet the inclusion criteria, but which were excluded, and explain why they were excluded. | Explained under first paragraph of ‘Results’. |
| Study characteristics | 17 | Cite each included study and present its characteristics. | Reported under under first paragraph of ‘Results’, Table 1 and Supplementary Table 4. |
| Risk of bias in studies | 18 | Present assessments of risk of bias for each included study. | Provided in Supplementary Figures 1 and 2. |
| Results of individual studies | 19 | For all outcomes, present, for each study: (a) summary statistics for each group (where appropriate) and (b) an effect estimate and its precision (e.g. confidence/credible interval), ideally using structured tables or plots. | Provided in Figures 2-6. |
| Results of syntheses | 20a | For each synthesis, briefly summarise the characteristics and risk of bias among contributing studies. | Reported in Table 1, Supplementary Table 3, Supplementary Figures 1 and 2 respectively. |
|  | 20b | Present results of all statistical syntheses conducted. If meta-analysis was done, present for each the summary estimate and its precision (e.g. confidence/credible interval) and measures of statistical heterogeneity. If comparing groups, describe the direction of the effect. | Reported under ‘Clinical Outcomes’ and ‘Hemodynamic Outcomes’ and depicted in Fig 2-6. |
|  | 20c | Present results of all investigations of possible causes of heterogeneity among study results. | Reported under ‘Results’ and provided in Supplementary Tables 6-8. |
|  | 20d | Present results of all sensitivity analyses conducted to assess the robustness of the synthesized results. | Reported under ‘Clinical Outcomes’ and ‘Hemodynamic Outcomes’. |
| Reporting biases | 21 | Present assessments of risk of bias due to missing results (arising from reporting biases) for each synthesis assessed. | No publication bias analyses were conducted due to the limited number of included studies, however, our explanation was provided under ‘Limitations’. |
| Certainty of evidence | 22 | Present assessments of certainty (or confidence) in the body of evidence for each outcome assessed. | Reported under ‘Clinical Outcomes’ and ‘Hemodynamic Outcomes’. |
| **DISCUSSION** | | |  |
| Discussion | 23a | Provide a general interpretation of the results in the context of other evidence. | Provided in ‘Discussion’. |
|  | 23b | Discuss any limitations of the evidence included in the review. | Provided in ‘Discussion’. |
|  | 23c | Discuss any limitations of the review processes used. | Provided in ‘Limitations’. |
|  | 23d | Discuss implications of the results for practice, policy, and future research. | Provided in ‘Discussion’. |
| **OTHER INFORMATION** | | |  |
| Registration and protocol | 24a | Provide registration information for the review, including register name and registration number, or state that the review was not registered. | Provided in first paragraph of ‘Methods’. |
|  | 24b | Indicate where the review protocol can be accessed, or state that a protocol was not prepared. | Provided in first paragraph of ‘Methods’. |
|  | 24c | Describe and explain any amendments to information provided at registration or in the protocol. | No amendments were made. |
| Support | 25 | Describe sources of financial or non-financial support for the review, and the role of the funders or sponsors in the review. | Provided in title page. |
| Competing interests | 26 | Declare any competing interests of review authors. | Provided in cover letter. |
| Availability of data, code and other materials | 27 | Report which of the following are publicly available and where they can be found: template data collection forms; data extracted from included studies; data used for all analyses; analytic code; any other materials used in the review. | Data were provided from the included studies and are available upon reasonable request from the corresponding author. |

*From:*  Page MJ, McKenzie JE, Bossuyt PM, Boutron I, Hoffmann TC, Mulrow CD, et al. The PRISMA 2020 statement: an updated guideline for reporting systematic reviews. BMJ 2021;372:n71. doi: 10.1136/bmj.n71

For more information, visit: <http://www.prisma-statement.org/>

**Supplementary Table 1.** Checklist of compliance with PRISMA guidelines.

| **Embase** | |
| --- | --- |
| **#1:**  **Patient** | ('Aortic Valve Stenosis'):ti,ab,kw OR (('aortic stenos*'):ti,ab,kw) OR (('Aortic Valve Stenos*'):ti,ab,kw) OR (('Aortic Valve Stenoses'):ti,ab,kw) OR (('Stenoses, Aortic Valve'):ti,ab,kw) OR (('Stenosis, Aortic Valve'):ti,ab,kw) OR (('Valve Stenoses, Aortic'):ti,ab,kw) OR (('Valve Stenosis, Aortic'):ti,ab,kw) OR (('Aortic Stenosis'):ti,ab,kw) OR (('Stenoses, Aortic'):ti,ab,kw) OR (('Stenosis, Aortic'):ti,ab,kw) OR (('low risk'):ti,ab,kw) OR ((low-risk):ti,ab,kw) OR (('low surgical risk'):ti,ab,kw) OR (('low-surgical risk'):ti,ab,kw) OR (('intermediate risk'):ti,ab,kw) OR ((intermediate-risk):ti,ab,kw) OR (('intermediate surgical risk'):ti,ab,kw) OR (('intermediate-surgical risk'):ti,ab,kw) OR (('Bicuspid Aortic Valve Disease'):ti,ab,kw) OR (('bicuspid valve'):ti,ab,kw) OR (('bicuspid aortic valve'):ti,ab,kw) OR ((BAV):ti,ab,kw) OR (('Aortic Valve, Bicuspid'):ti,ab,kw) OR (('Bicuspid Aortic Valve'):ti,ab,kw) OR (('Bicuspid Aortic Valves'):ti,ab,kw) OR (('Valve, Bicuspid Aortic'):ti,ab,kw) OR (('Purely Bicuspid Aortic Valve'):ti,ab,kw) OR (('Single Raphe Bicuspid Aortic Valve'):ti,ab,kw) OR (('Bicuspid Aortic Valve'):ti,ab,kw) OR (('Single Raphe'):ti,ab,kw) OR (('Aortic Valve Disease 1'):ti,ab,kw) OR (('Two-Raphe Bicuspid Aortic Valve'):ti,ab,kw) OR (('Double Raphe Bicuspid Aortic Valve'):ti,ab,kw) OR (('Two Raphe Bicuspid Aortic Valve'):ti,ab,kw) OR (('Bicuspid Aortic Valve, Two-Raphe'):ti,ab,kw) OR (('Bicuspid Aortic Valve, Two Raphe'):ti,ab,kw) OR (('systolic dysfunction'):ti,ab,kw) OR (('Ventricular Dysfunction, Left'):ti,ab,kw) OR (('Ventricular Dysfunction'):ti,ab,kw) OR (('Dysfunction, Ventricular'):ti,ab,kw) OR (('Dysfunctions, Ventricular'):ti,ab,kw) OR (('Ventricular Dysfunctions'):ti,ab,kw) OR (('Dysfunction, Left Ventricular'):ti,ab,kw) OR (('Left Ventricular Dysfunctions'):ti,ab,kw) OR (('LV Dysfunction'):ti,ab,kw) OR (('Dysfunction, LV'):ti,ab,kw) OR (('LV Dysfunctions'):ti,ab,kw) OR (('Left Ventricular Dysfunction'):ti,ab,kw) OR (('Left Ventricular Systolic Dysfunction'):ti,ab,kw) OR (('LV Systolic Dysfunction'):ti,ab,kw) OR (('Dysfunction, LV Systolic'):ti,ab,kw) OR (('LV Systolic Dysfunctions'):ti,ab,kw) OR (('Systolic Dysfunction, LV'):ti,ab,kw) OR (('calcified aortic valve'):ti,ab,kw) OR (('calcific aortic valve'):ti,ab,kw) OR (('aortic valve calcification'):ti,ab,kw) OR (('Aortic Stenosis, Calcific'):ti,ab,kw) OR (('aortic valve calcific*'):ti,ab,kw) OR (('Aortic Valve, Calcification'):ti,ab,kw) OR (('aortic valve degeneration'):ti,ab,kw) OR (('aortic valve degenerat*'):ti,ab,kw) |
| **#2:**  **Intervention** | ('Aortic Valve Stenosis'):ti,ab,kw OR ((tavr):ti,ab,kw) OR (('Transcatheter Aortic Valve Implantation'):ti,ab,kw) OR ((tavi):ti,ab,kw) OR (('percutaneous aortic valve replacement'):ti,ab,kw) OR (('transfemoral aortic valve replacement'):ti,ab,kw) |
| **#3:**  **comparison** | ('self expanding valve'):ti,ab,kw OR ((sev):ti,ab,kw) OR (('self expand* valve'):ti,ab,kw) OR (('Self expanding valve*'):ti,ab,kw) OR (('Self expandable valve*'):ti,ab,kw) OR (('Self expanded valve*'):ti,ab,kw) OR (('self expand*'):ti,ab,kw) OR (('balloon expanding valve'):ti,ab,kw) OR ((bev):ti,ab,kw) OR (('balloon expand*'):ti,ab,kw) OR (('Balloon expandable valve*'):ti,ab,kw) OR (('Balloon expanding valve*'):ti,ab,kw) OR (('Balloon expanded valve*'):ti,ab,kw) OR (('New generation valve*'):ti,ab,kw) OR (('second generation valve*'):ti,ab,kw) OR (('third generation valve*'):ti,ab,kw) OR (('next generation valve*'):ti,ab,kw) OR (('new generation device*'):ti,ab,kw) OR (('next generation device*'):ti,ab,kw) OR (('latest generation device*'):ti,ab,kw) OR (('second generation device*'):ti,ab,kw) OR (('third generation device*'):ti,ab,kw) OR (('Core Valve'):ti,ab,kw) OR ((CoreValve):ti,ab,kw) OR ((Evolut):ti,ab,kw) OR (('Evolut R'):ti,ab,kw) OR (('Evolut PRO'):ti,ab,kw) OR ((Sapien):ti,ab,kw) OR (('sapien XT'):ti,ab,kw) OR (('Sapien 3'):ti,ab,kw) OR (('Sapien 3 Ultra'):ti,ab,kw) OR ((Acurate):ti,ab,kw) OR ((AcurateNeo):ti,ab,kw) OR (('Conformite Eurioeene'):ti,ab,kw) OR (('Jena valve'):ti,ab,kw) OR ((centera):ti,ab,kw) OR (('Venus A-valve'):ti,ab,kw) OR ((Portico):ti,ab,kw) OR ((Allegra):ti,ab,kw) OR ((myval):ti,ab,kw) |
| **#4:**  **study** | randomised OR 'randomized controlled trial' OR (clinical AND trial) OR rct OR (randomized AND controlled AND trial) |
| **Total** | #1 AND (#2 OR #3) AND #4 |

| **PubMed** | |
| --- | --- |
| **#1:**  **Patient** | "aortic valve stenosis"[MeSH Terms] OR "aortic stenos*"[Title/Abstract] OR "aortic valve stenos*"[Title/Abstract] OR "aortic valve stenoses"[Title/Abstract] OR "stenoses aortic valve"[Title/Abstract] OR "stenosis aortic valve"[Title/Abstract] OR "valve stenoses aortic"[Title/Abstract] OR "valve stenosis aortic"[Title/Abstract] OR "aortic stenosis"[Title/Abstract] OR "stenoses aortic"[Title/Abstract] OR "stenosis aortic"[Title/Abstract] OR "aortic valve stenosis"[Title/Abstract] OR "aortic valve/surgery"[MeSH Terms] OR "Bicuspid Aortic Valve Disease"[MeSH Terms] OR ("Bicuspid"[Title/Abstract] OR "bicuspid valve"[Title/Abstract] OR "bicuspid aortic valve"[Title/Abstract] OR "BAV"[Title/Abstract] OR "aortic valve bicuspid"[Title/Abstract] OR "bicuspid aortic valve"[Title/Abstract] OR "bicuspid aortic valves"[Title/Abstract] OR "valve bicuspid aortic"[Title/Abstract] OR "purely bicuspid aortic valve"[Title/Abstract] OR ((("single person"[MeSH Terms] OR ("Single"[All Fields] AND "person"[All Fields]) OR "single person"[All Fields] OR "Single"[All Fields] OR "singles"[All Fields]) AND ("Raphe"[All Fields] OR "raphes"[All Fields])) AND "bicuspid aortic valve"[Title/Abstract]) OR (("Bicuspid Aortic Valve Disease"[MeSH Terms] OR ("Bicuspid"[All Fields] AND "Aortic"[All Fields] AND "Valve"[All Fields] AND "Disease"[All Fields]) OR "Bicuspid Aortic Valve Disease"[All Fields] OR ("Bicuspid"[All Fields] AND "Aortic"[All Fields] AND "Valve"[All Fields]) OR "bicuspid aortic valve"[All Fields]) AND "single raphe"[Title/Abstract]) OR "aortic valve disease 1"[Title/Abstract] OR ("Two-Raphe"[All Fields] AND "bicuspid aortic valve"[Title/Abstract]) OR ((("double"[All Fields] OR "doubled"[All Fields] OR "doubles"[All Fields] OR "doubling"[All Fields] OR "doublings"[All Fields]) AND ("Raphe"[All Fields] OR "raphes"[All Fields])) AND "bicuspid aortic valve"[Title/Abstract]) OR (("Two"[All Fields] AND ("Raphe"[All Fields] OR "raphes"[All Fields])) AND "bicuspid aortic valve"[Title/Abstract]) OR (("Bicuspid Aortic Valve Disease"[MeSH Terms] OR ("Bicuspid"[All Fields] AND "Aortic"[All Fields] AND "Valve"[All Fields] AND "Disease"[All Fields]) OR "Bicuspid Aortic Valve Disease"[All Fields] OR ("Bicuspid"[All Fields] AND "Aortic"[All Fields] AND "Valve"[All Fields]) OR "bicuspid aortic valve"[All Fields]) AND "Two-Raphe"[Title/Abstract]) OR (("Bicuspid Aortic Valve Disease"[MeSH Terms] OR ("Bicuspid"[All Fields] AND "Aortic"[All Fields] AND "Valve"[All Fields] AND "Disease"[All Fields]) OR "Bicuspid Aortic Valve Disease"[All Fields] OR ("Bicuspid"[All Fields] AND "Aortic"[All Fields] AND "Valve"[All Fields]) OR "bicuspid aortic valve"[All Fields]) AND "Two-Raphe"[Title/Abstract])) OR "Ventricular Dysfunction"[MeSH Terms] OR "ventricular dysfunction, left"[MeSH Terms] OR "systolic dysfunction"[Title/Abstract] OR "dysfunction ventricular"[Title/Abstract] OR "dysfunctions ventricular"[Title/Abstract] OR "ventricular dysfunctions"[Title/Abstract] OR "dysfunction left ventricular"[Title/Abstract] OR "left ventricular dysfunctions"[Title/Abstract] OR "lv dysfunction"[Title/Abstract] OR "dysfunction lv"[Title/Abstract] OR "lv dysfunctions"[Title/Abstract] OR "left ventricular dysfunction"[Title/Abstract] OR "left ventricular systolic dysfunction"[Title/Abstract] OR "lv systolic dysfunction"[Title/Abstract] OR "dysfunction lv systolic"[Title/Abstract] OR "lv systolic dysfunctions"[Title/Abstract] OR "systolic dysfunction lv"[Title/Abstract] OR "low-risk"[Title/Abstract] OR "low-risk"[Title/Abstract] OR "low surgical risk"[Title/Abstract] OR "low surgical risk"[Title/Abstract] OR "intermediate-risk"[Title/Abstract] OR "intermediate-risk"[Title/Abstract] OR "intermediate surgical risk"[Title/Abstract] OR "intermediate surgical risk"[Title/Abstract] OR "aortic valve degeneration"[Title/Abstract] OR "aortic valve degenerat*"[Title/Abstract] OR "calcified aortic valve"[Title/Abstract] OR "calcific aortic valve"[Title/Abstract] OR "aortic valve calcification"[Title/Abstract] OR "aortic stenosis calcific"[Title/Abstract] OR "aortic valve calcific*"[Title/Abstract] |
| **#2:**  **Intervention** | "Transcatheter Aortic Valve Replacement"[MeSH Terms] OR "tavr"[Title/Abstract] OR "transcatheter aortic valve implantation"[Title/Abstract] OR "tavi"[Title/Abstract] OR "percutaneous aortic valve replacement"[Title/Abstract] OR "transfemoral aortic valve replacement"[Title/Abstract] OR "Transcatheter Aortic Valve Replacement"[Title/Abstract] |
| **#3:**  **comparison** | "self expanding valve"[Title/Abstract] OR "sev"[Title/Abstract] OR ((("ego"[MeSH Terms] OR "ego"[All Fields] OR "Self"[All Fields]) AND "expand*"[All Fields]) AND "Valve"[Title/Abstract]) OR "self expanding valve*"[Title/Abstract] OR "self expandable valve*"[Title/Abstract] OR (("ego"[MeSH Terms] OR "ego"[All Fields] OR "Self"[All Fields]) AND "expanded valve*"[Title/Abstract]) OR "self expand*"[Title/Abstract] OR "balloon expanding valve"[Title/Abstract] OR "bev"[Title/Abstract] OR "balloon expand*"[Title/Abstract] OR "balloon expandable valve*"[Title/Abstract] OR "balloon expanding valve*"[Title/Abstract] OR (("Balloon"[All Fields] OR "balloon s"[All Fields] OR "balloons"[All Fields]) AND "expanded valve*"[Title/Abstract]) OR "new generation valve*"[Title/Abstract] OR "second generation valve*"[Title/Abstract] OR "third generation valve*"[Title/Abstract] OR "next generation valve*"[Title/Abstract] OR "new generation device*"[Title/Abstract] OR "next generation device*"[Title/Abstract] OR "latest generation device*"[Title/Abstract] OR "second generation device*"[Title/Abstract] OR "third generation device*"[Title/Abstract] OR "core valve"[Title/Abstract] OR "CoreValve"[Title/Abstract] OR "Evolut"[Title/Abstract] OR "evolut r"[Title/Abstract] OR "evolut pro"[Title/Abstract] OR "Sapien"[Title/Abstract] OR "sapien xt"[Title/Abstract] OR "sapien 3"[Title/Abstract] OR "sapien 3 ultra"[Title/Abstract] OR "Acurate"[Title/Abstract] OR "AcurateNeo"[Title/Abstract] OR "jena valve"[Title/Abstract] OR "centera"[Title/Abstract] OR "venus a valve"[Title/Abstract] OR "Portico"[Title/Abstract] OR "Allegra"[Title/Abstract] OR "myval"[Title/Abstract] |
| **#4:**  **study** | "random allocation"[MeSH Terms] OR ("random"[All Fields] AND "allocation"[All Fields]) OR "random allocation"[All Fields] OR "randomization"[All Fields] OR "randomized"[All Fields] OR "random"[All Fields] OR "randomisation"[All Fields] OR "randomisations"[All Fields] OR "randomise"[All Fields] OR "randomised"[All Fields] OR "randomising"[All Fields] OR "randomizations"[All Fields] OR "randomize"[All Fields] OR "randomizes"[All Fields] OR "randomizing"[All Fields] OR "randomness"[All Fields] OR "randoms"[All Fields] OR ("random allocation"[MeSH Terms] OR ("random"[All Fields] AND "allocation"[All Fields]) OR "random allocation"[All Fields] OR "randomization"[All Fields] OR "randomized"[All Fields] OR "random"[All Fields] OR "randomisation"[All Fields] OR "randomisations"[All Fields] OR "randomise"[All Fields] OR "randomised"[All Fields] OR "randomising"[All Fields] OR "randomizations"[All Fields] OR "randomize"[All Fields] OR "randomizes"[All Fields] OR "randomizing"[All Fields] OR "randomness"[All Fields] OR "randoms"[All Fields]) OR ("Clinical Trial"[Publication Type] OR "clinical trials as topic"[MeSH Terms] OR "Clinical Trial"[All Fields]) OR "RCT"[All Fields] OR ("Clinical Trial"[Publication Type] OR "Randomized Controlled Trial"[Publication Type]) |
| **Total** | #1 AND (#2 OR #3) AND #4 |

| **Cochrane** | |
| --- | --- |
| **#1:**  **Patient** | MeSH descriptor: [Aortic Valve] explode all trees OR MeSH descriptor: [Aortic Valve Stenosis] explode all trees OR (aortic stenos*):ti,ab,kw OR (Aortic Valve Stenos*):ti,ab,kw OR (Aortic Valve Stenoses):ti,ab,kw OR (Stenoses, Aortic Valve):ti,ab,kw OR (Stenosis, Aortic Valve):ti,ab,kw OR (Valve Stenoses, Aortic):ti,ab,kw OR (Valve Stenosis, Aortic):ti,ab,kw OR (Aortic Stenosis):ti,ab,kw OR (Stenoses, Aortic):ti,ab,kw OR (Stenosis, Aortic):ti,ab,kw OR (low risk):ti,ab,kw OR (low-risk):ti,ab,kw OR (low surgical risk):ti,ab,kw OR (low-surgical risk):ti,ab,kw OR (intermediate risk):ti,ab,kw OR (intermediate-risk):ti,ab,kw OR (intermediate surgical risk):ti,ab,kw OR (intermediate-surgical risk):ti,ab,kw OR MeSH descriptor: [Bicuspid Aortic Valve Disease] explode all trees OR (bicuspid):ti,ab,kw OR (bicuspid valve):ti,ab,kw OR (bicuspid aortic valve):ti,ab,kw OR (BAV):ti,ab,kw OR (Aortic Valve, Bicuspid):ti,ab,kw OR (Bicuspid Aortic Valve):ti,ab,kw OR (Bicuspid Aortic Valves):ti,ab,kw OR (Valve, Bicuspid Aortic):ti,ab,kw OR (Purely Bicuspid Aortic Valve):ti,ab,kw OR (Single Raphe Bicuspid Aortic Valve):ti,ab,kw OR (Bicuspid Aortic Valve, Single Raphe):ti,ab,kw OR (Aortic Valve Disease 1):ti,ab,kw OR (Two-Raphe Bicuspid Aortic Valve):ti,ab,kw OR (Double Raphe Bicuspid Aortic Valve):ti,ab,kw OR (Two Raphe Bicuspid Aortic Valve):ti,ab,kw OR (Bicuspid Aortic Valve, Two-Raphe):ti,ab,kw OR (Bicuspid Aortic Valve, Two Raphe):ti,ab,kw OR MeSH descriptor: [Ventricular Dysfunction] explode all trees OR MeSH descriptor: [Ventricular Dysfunction, Left] explode all trees OR (systolic dysfunction):ti,ab,kw OR (Dysfunction, Ventricular):ti,ab,kw OR (Dysfunctions, Ventricular):ti,ab,kw OR (Ventricular Dysfunctions):ti,ab,kw OR (Dysfunction, Left Ventricular):ti,ab,kw OR (Left Ventricular Dysfunctions):ti,ab,kw OR (LV Dysfunction):ti,ab,kw OR (Dysfunction, LV):ti,ab,kw OR (LV Dysfunctions):ti,ab,kw OR (Left Ventricular Dysfunction):ti,ab,kw OR (Left Ventricular Systolic Dysfunction):ti,ab,kw OR (LV Systolic Dysfunction):ti,ab,kw OR (Dysfunction, LV Systolic):ti,ab,kw OR (LV Systolic Dysfunctions):ti,ab,kw OR (Systolic Dysfunction, LV):ti,ab,kw OR (calcified aortic valve):ti,ab,kw OR (calcific aortic valve):ti,ab,kw OR (aortic valve calcification):ti,ab,kw OR (Aortic Stenosis, Calcific):ti,ab,kw OR (aortic valve calcific*):ti,ab,kw OR (aortic valve degeneration):ti,ab,kw OR (aortic valve degenerat*):ti,ab,kw |
| **#2:**  **Intervention** | MeSH descriptor: [Transcatheter Aortic Valve Replacement] explode all trees OR (tavr):ti,ab,kw OR (Transcatheter Aortic Valve Implantation):ti,ab,kw OR (tavi):ti,ab,kw OR (percutaneous aortic valve replacement):ti,ab,kw OR (transfemoral aortic valve replacement):ti,ab,kw OR (Transcatheter Aortic Valve Replacement):ti,ab,kw |
| **#3:**  **comparison** | (self expanding valve):ti,ab,kw OR (sev):ti,ab,kw OR (self expand* valve):ti,ab,kw OR (Self expanding valve*):ti,ab,kw OR (Self expandable valve*):ti,ab,kw OR (Self expanded valve*):ti,ab,kw OR (self expand*):ti,ab,kw OR (balloon expanding valve):ti,ab,kw OR (bev):ti,ab,kw OR (balloon expand*):ti,ab,kw OR (Balloon expandable valve*):ti,ab,kw OR (Balloon expanding valve*):ti,ab,kw OR (Balloon expanded valve*):ti,ab,kw OR (New generation valve*):ti,ab,kw OR (second generation valve*):ti,ab,kw OR (third generation valve*):ti,ab,kw OR (next generation valve*):ti,ab,kw OR (new generation device*):ti,ab,kw OR (next generation device*):ti,ab,kw OR (latest generation device*):ti,ab,kw OR (second generation device*):ti,ab,kw OR (third generation device*):ti,ab,kw OR (Core Valve):ti,ab,kw OR (CoreValve):ti,ab,kw OR (Evolut):ti,ab,kw OR (Evolut R):ti,ab,kw OR (Evolut PRO):ti,ab,kw OR (Sapien):ti,ab,kw OR (sapien XT):ti,ab,kw OR (Sapien 3):ti,ab,kw OR (Sapien 3 Ultra):ti,ab,kw OR (Acurate):ti,ab,kw OR (AcurateNeo):ti,ab,kw OR (Conformite Eurioeene):ti,ab,kw OR (Jena valve):ti,ab,kw OR (centera):ti,ab,kw OR (Venus A-valve):ti,ab,kw OR (Portico):ti,ab,kw OR (Allegra):ti,ab,kw OR (myval):ti,ab,kw |
| **#4:**  **study** | MeSH descriptor: [Clinical Trial] explode all trees OR MeSH descriptor: [Randomized Controlled Trial] explode all trees OR (randomised) OR (randomized) OR (clinical trial) OR (RCT) |
| **Total** | #1 AND (#2 OR #3) AND #4 |

| **Scopus** | |
| --- | --- |
| **#1:**  **Patient** | ( TITLE-ABS-KEY ( aortic AND valve AND stenosis ) OR TITLE-ABS-KEY ( aortic AND stenos* ) OR TITLE-ABS-KEY ( aortic AND valve AND stenos* ) OR TITLE-ABS-KEY ( aortic AND valve AND stenoses ) OR TITLE-ABS-KEY ( stenoses, AND aortic AND valve ) OR TITLE-ABS-KEY ( stenosis, AND aortic AND valve ) OR TITLE-ABS-KEY ( valve AND stenoses, AND aortic ) OR TITLE-ABS-KEY ( valve AND stenosis, AND aortic ) OR TITLE-ABS-KEY ( aortic AND stenosis ) OR TITLE-ABS-KEY ( stenoses, AND aortic ) OR TITLE-ABS-KEY ( stenosis, AND aortic ) OR TITLE-ABS-KEY ( aortic AND valve AND surgery ) ) OR ( TITLE-ABS-KEY ( low AND risk ) OR TITLE-ABS-KEY ( low-risk ) OR TITLE-ABS-KEY ( low AND surgical AND risk ) OR TITLE-ABS-KEY ( low-surgical AND risk ) OR TITLE-ABS-KEY ( intermediate AND risk ) OR TITLE-ABS-KEY ( intermediate-risk ) OR TITLE-ABS-KEY ( intermediate AND surgical AND risk ) OR TITLE-ABS-KEY ( intermediate-surgical AND risk ) ) OR ( TITLE-ABS-KEY ( bicuspid AND aortic AND valve AND disease ) OR TITLE-ABS-KEY ( bicuspid ) OR TITLE-ABS-KEY ( bicuspid AND valve ) OR TITLE-ABS-KEY ( bicuspid AND aortic AND valve ) OR TITLE-ABS-KEY ( bav ) OR TITLE-ABS-KEY ( aortic AND valve, AND bicuspid ) OR TITLE-ABS-KEY ( bicuspid AND aortic AND valve ) OR TITLE-ABS-KEY ( bicuspid AND aortic AND valves ) OR TITLE-ABS-KEY ( valve, AND bicuspid AND aortic ) OR TITLE-ABS-KEY ( purely AND bicuspid AND aortic AND valve ) OR TITLE-ABS-KEY ( single AND raphe AND bicuspid AND aortic AND valve ) OR TITLE-ABS-KEY ( bicuspid AND aortic AND valve, AND single AND raphe ) OR TITLE-ABS-KEY ( aortic AND valve AND disease 1 ) OR TITLE-ABS-KEY ( two-raphe AND bicuspid AND aortic AND valve ) OR TITLE-ABS-KEY ( double AND raphe AND bicuspid AND aortic AND valve ) OR TITLE-ABS-KEY ( two AND raphe AND bicuspid AND aortic AND valve ) OR TITLE-ABS-KEY ( bicuspid AND aortic AND valve, AND two-raphe ) OR TITLE-ABS-KEY ( bicuspid AND aortic AND valve, AND two AND raphe ) ) OR ( TITLE-ABS-KEY ( systolic AND dysfunction ) OR TITLE-ABS-KEY ( ventricular AND dysfunction, AND left ) OR TITLE-ABS-KEY ( ventricular AND dysfunction ) OR TITLE-ABS-KEY ( dysfunction, AND ventricular ) OR TITLE-ABS-KEY ( dysfunctions, AND ventricular ) OR TITLE-ABS-KEY ( ventricular AND dysfunctions ) OR TITLE-ABS-KEY ( dysfunction, AND left AND ventricular ) OR TITLE-ABS-KEY ( left AND ventricular AND dysfunctions ) OR TITLE-ABS-KEY ( lv AND dysfunction ) OR TITLE-ABS-KEY ( dysfunction, AND lv ) OR TITLE-ABS-KEY ( lv AND dysfunctions ) OR TITLE-ABS-KEY ( left AND ventricular AND dysfunction ) OR TITLE-ABS-KEY ( left AND ventricular AND systolic AND dysfunction ) OR TITLE-ABS-KEY ( lv AND systolic AND dysfunction ) OR TITLE-ABS-KEY ( dysfunction, AND lv AND systolic ) OR TITLE-ABS-KEY ( lv AND systolic AND dysfunctions ) OR TITLE-ABS-KEY ( systolic AND dysfunction, AND lv ) ) OR ( TITLE-ABS-KEY ( calcified AND aortic AND valve ) OR TITLE-ABS-KEY ( calcific AND aortic AND valve ) OR TITLE-ABS-KEY ( aortic AND valve AND calcification ) OR TITLE-ABS-KEY ( aortic AND stenosis, AND calcific ) OR TITLE-ABS-KEY ( aortic AND valve AND calcific* ) ) OR ( TITLE-ABS-KEY ( aortic AND valve AND degeneration ) OR TITLE-ABS-KEY ( aortic AND valve AND degenerat* )) |
| **#2:**  **Intervention** | ( TITLE-ABS-KEY ( transcatheter AND aortic AND valve AND replacement ) OR TITLE-ABS-KEY ( tavr ) OR TITLE-ABS-KEY ( transcatheter AND aortic AND valve AND implantation ) OR TITLE-ABS-KEY ( tavi ) OR TITLE-ABS-KEY ( percutaneous AND aortic AND valve AND replacement ) OR TITLE-ABS-KEY ( transfemoral AND aortic AND valve AND replacement )) |
| **#3:**  **comparison** | (TITLE-ABS-KEY ( self AND expanding AND valve ) OR TITLE-ABS-KEY ( sev ) OR TITLE-ABS-KEY ( self AND expand* AND valve ) OR TITLE-ABS-KEY ( self AND expanding AND valve* ) OR TITLE-ABS-KEY ( self AND expandable AND valve* ) OR TITLE-ABS-KEY ( self AND expanded AND valve* ) OR TITLE-ABS-KEY ( self AND expand* ) OR TITLE-ABS-KEY ( balloon AND expanding AND valve ) OR TITLE-ABS-KEY ( bev ) OR TITLE-ABS-KEY ( balloon AND expand* ) OR TITLE-ABS-KEY ( balloon AND expandable AND valve* ) OR TITLE-ABS-KEY ( balloon AND expanding AND valve* ) OR TITLE-ABS-KEY ( balloon AND expanded AND valve* ) OR TITLE-ABS-KEY ( new AND generation AND valve* ) OR TITLE-ABS-KEY ( second AND generation AND valve* ) OR TITLE-ABS-KEY ( third AND generation AND valve* ) OR TITLE-ABS-KEY ( next AND generation AND valve* ) OR TITLE-ABS-KEY ( new AND generation AND device* ) OR TITLE-ABS-KEY ( next AND generation AND device* ) OR TITLE-ABS-KEY ( latest AND generation AND device* ) OR TITLE-ABS-KEY ( second AND generation AND device* ) OR TITLE-ABS-KEY ( third AND generation AND device* ) OR TITLE-ABS-KEY ( core AND valve ) OR TITLE-ABS-KEY ( corevalve ) OR TITLE-ABS-KEY ( evolut ) OR TITLE-ABS-KEY ( evolut AND r ) OR TITLE-ABS-KEY ( evolut AND pro ) OR TITLE-ABS-KEY ( sapien ) OR TITLE-ABS-KEY ( sapien AND xt ) OR TITLE-ABS-KEY ( sapien 3 ) OR TITLE-ABS-KEY ( sapien 3 ultra ) OR TITLE-ABS-KEY ( acurate ) OR TITLE-ABS-KEY ( acurateneo ) OR TITLE-ABS-KEY ( conformite AND eurioeene ) OR TITLE-ABS-KEY ( jena AND valve ) OR TITLE-ABS-KEY ( centera ) OR TITLE-ABS-KEY ( venus AND a-valve ) OR TITLE-ABS-KEY ( portico ) OR TITLE-ABS-KEY ( allegra ) OR TITLE-ABS-KEY ( myval ) ) |
| **#4:**  **study** | ( TITLE-ABS-KEY ( randomized ) OR TITLE-ABS-KEY ( randomised ) OR TITLE-ABS-KEY ( clinical AND trial ) OR TITLE-ABS-KEY ( rct ) OR TITLE-ABS-KEY ( randomized AND controlled AND trial ) ) |
| **Total** | #1 AND (#2 OR #3) AND #4 |

**Supplementary Table 2.** Search strategy

| **Variable group** | **Variable** |
| --- | --- |
| Study characteristics | Publication year, trial name, trial location, surgical risk of included patients (low, intermediate or high), anatomical characteristics of the aortic annulus (small aortic annulus or no small aortic annulus), brand of BEV and SEV used in the trial. |
| Study population characteristics | Sample sizes of the BEV and SEV groups, patients demographics (mean age, sex distribution, and their background diseases), baseline Euroscore and STS score, and baseline hemodynamic characteristics (left ventricular ejection fraction, mean trans-valvular pressure gradient, and aortic valve area). |
| Procedural outcomes | Contrast volume, procedure duration, fluoroscopy time, procedure success rate, second valve implantation and number of pre-dilatations and post-dilatation procedures of each valve type. |
| Clinical outcomes | Major adverse cardiovascular events, all-cause mortality, cardiovascular mortality, stroke (total, disabling and non-disabling), heart failure hospitalization, new atrial fibrillation, PPI, clinical valve thrombosis, endocarditis, major bleeding event, major cardiovascular events, and acute kidney injury. |
| Hemodynamic outcomes | EOA (cm^2^), mild PVL, moderate to severe PVL, and mean trans-valvular pressure gradient (mmHg) |

**Supplementary Table 3.** Extracted data from each study.

BEV, Balloon-expandable valves; EOA, Effective orifice area; PPI, permanent pacemaker implantation; PVL, Paravalvular leak; SEV, Self-expanding valves; STS, Society of Thoracic Surgeons

| **Clinical trial** | **Valve type** | **Previous medical history, (N)** | | | | | | | | | | | | **Predicted mortality (%)** | | | **Echocardiographic characteristics** | | | |
| --- | --- | --- | --- | --- | --- | --- | --- | --- | --- | --- | --- | --- | --- | --- | --- | --- | --- | --- | --- | --- |
|  |  | **DM** | **HTN** | **CAD** | **COPD** | **NYHA 3/4** | **PPM** | **MI** | **PCI** | **Stroke** | **AF** | **CKD** | **Euroscore-median(IQR)** | | **STS Score** | **LVEF, (%)** | | **AV mean gradient-mm Hg** | **AV area-mm** |  |
| **BRAVO 3** | SEV | 71 | NA | 151 | 65 | 32 | NA | 48 | NA | 32 | 94 | 156 | NA | | NA | NA | | NA | NA |  |
|  | BEV | 164 | NA | 245 | 90 | 39 | NA | 64 | NA | 48 | 192 | 247 | NA | | NA | NA | | NA | NA |  |
| **CHOICE** | SEV | 32 | 110 | 79 | 24 | 98 | 9 | 16 | 51 | 22 | 29 | 10 | 4.4 (2.9-7.9) | | 6.2 ± 3.9 | 54.9 ± 11.9 | | 4 3± 13.9 | 0.7± 0.2 |  |
|  | BEV | 38 | 111 | 73 | 27 | 97 | 7 | 14 | 44 | 26 | 39 | 7 | 4.6 (2.9-7.9) | | 5.6 ± 2.9 | 52.5 ± 13.8 | | 43.3 ± 15.4 | 0.7 ± 0.2 |  |
| **ELECT** | SEV | 11 | 15 | 10 | 7 | 13 | 0 | 7 | 9 | 4 | 7 | NA | NA | | NA | 51 ± 11 | | 44 ± 20 | NA |  |
|  | BEV | 6 | 23 | 17 | 1 | 16 | 2 | 7 | 10 | 5 | 10 | NA | NA | | NA | 53 ± 10 | | 42 ± 13 | NA |  |
| **LYTEN** | SEV | 20 | 47 | NA | NA | NA | NA | NA | NA | NA | 16 | 22 | NA | | 4.9 | 57 ± 12 | | 41 ± 14 | 0.95 ± 0.36 |  |
|  | BEV | 18 | 42 | NA | NA | NA | NA | NA | NA | NA | 12 | 24 | NA | | 5.9 | 58 ± 12 | | 43 ± 16 | 0.93 ± 0.36 |  |
| **PORTICO IDE** | SEV | 143 | 358 | 261 | 156 | 267 | 55 | 54 | 106 | 28 | 122 | 94 | 6.4 ± 3.4 | | 6.3 ± 3.4 | 57.3 ± 11.3 | | 0.68 ± 0.17 | 0.68 ± 0.17 |  |
|  | BEV | 73 | 184 | 150 | 81 | 151 | 39 | 20 | 59 | 32 | 81 | 44 | 6.8 ± 5.9 | | 6.2 ± 3.4 | 57.1 ± 10.8 | | 46.7 ± 11.7 | 0.68 ±0.16 |  |
| **SOLVE-TAVI** | SEV | 79 | 193 | 127 | 30 | 141 | 24 | 19 | 84 | 25 | 103 | 177 | 4.1 (2.5-7.5) | | 4.9 (2.9-9.9) | NA | | 38.5 (30-50.5) | 0.7 (0.6-0.9) |  |
|  | BEV | 68 | 204 | 116 | 29 | 145 | 23 | 22 | 79 | 26 | 93 | 184 | 3.8 (2.4-6.1) | | 4.7 (3.1-9.4) | NA | | 37 (26.5-47.5) | 0.8 (0.6-0.9) |  |
| **SCOPE 1** | SEV | 108 | 341 | 218 | 33 | 287 | 43 | 39 | 117 | 47 | 133 | 15 | NA | | 3.4 (2.6-5.2) | 57.1 ± 10.7 | | 41.5 ± 15.1 | 0.7 ± 0.2 |  |
|  | BEV | 116 | 333 | 219 | 44 | 268 | 36 | 47 | 126 | 47 | 136 | 17 | NA | | 3.7 (2.5-4.9) | 56.1 ± 11.1 | | 42.9 ± 17.2 | 0.7 ± 0.2 |  |
| **Elnaggar,2022** | SEV | 15 | 39 | 32 | 5 | 44 | 5 | NA | 21 | 3 | 25 | NA | NA | | 3.8 ± 2.6 | 48.4 ± 11.7 | | 42.3 ± 7.7 | 0.9 ± 0.7 |  |
|  | BEV | 22 | 49 | 27 | 14 | 50 | 4 | NA | 22 | 3 | 28 | NA | NA | | 3.5 ± 2.2 | 50.9 ± 11.7 | | 42.8 ± 9.9 | 0.9 ± 0.2 |  |
| **SMART** | SEV | 104 | 293 | 125 | 61 | 154 | 30 | 19 | 60 | 42 | 69 | NA | NA | | 3.3 ± 1.9 | 61.6 ± 7.6 | | NA | NA |  |
|  | BEV | 123 | 313 | 148 | 62 | 144 | 25 | 29 | 84 | 41 | 65 | NA | NA | | 3.2 ± 1.7 | 61.2 ± 8.7 | | NA | NA |  |
| **LANDMARK** | SEV | NA | NA | NA | NA | NA | NA | NA | NA | NA | NA | NA | NA | | NA | NA | | NA | NA |  |
|  | BEV | 50 | 256 | 55 | 42 | 206 | 11 | 26 | 30 | 13 | 94 | 53 | NA | | 2·6 (1·7–4·0) | 58·3 ± 10·7 | | 39·9 ±14 | 0·7 ± 0·2 |  |

**Supplementary Table 4.** Baseline and echocardiographic characteristics

Data are presented as number, median (IQR), or mean± SD. AF: Atrial fibrillation; AV: Aortic valve; BEV: Balloon-expandable valves; CAD: Coronary artery disease; CKD: Chronic Kidney Disease; COPD: Chronic obstructive pulmonary disease; DM: Diabetes; HTN: Hypertension; IQR: Interquartile range; LVEF: Left ventricular ejection fraction; MI: Myocardial infarction; NYHA: New York Heart Association; PCI: Percutaneous coronary interventions; PPM: Permanent pacemaker; SEV: Self-expanding valves; STS: Society of Thoracic Surgeons

| **Endpoint** | **No. of studies** | **Study design** | **Quality of evidence** | | | | | **Summary of findings** | | | **Quality** |
| --- | --- | --- | --- | --- | --- | --- | --- | --- | --- | --- | --- |
|  |  |  | Risk of bias | Inconsistency | Indirectness | Imprecision | Publications bias | RR/MD (95% CI) | P-value | Plausible  confounding |  |
| **All-cause mortality** | | | | | | | | | | | |
| Short-term | 7 | RCT | Not serious | Not serious | Not serious | Serious ^b^ | Not serious | 0.54 (0.35-0.81) | 0.003 | Not plausible | High |
| Mid-term | 7 | RCT | Not serious | Not serious | Not serious | Serious ^b^ | Not serious | 0.89 (0.69 – 1.2) | 0.4 | Not plausible | Moderate |
| Long-term | 3 | RCT | Not serious | Serious ^a^ | Not serious | Serious ^b^ | Not serious | 0.95 (0.72 – 1.25) | 0.7 | Not plausible | Low |
| **Cardiovascular mortality** | | | | | | | | | | | |
| Short-term | 7 | RCT | Not serious | Not serious | Not serious | Serious ^b^ | Not serious | 0.56 (0.36-0.87) | 0.01 | Not plausible | High |
| Mid-term | 6 | RCT | Not serious | Not serious | Not serious | Serious ^b^ | Not serious | 0.87 (0.59 – 1.29) | 0.5 | Not plausible | Moderate |
| Long-term | 3 | RCT | Not serious | Serious ^a^ | Not serious | Serious ^b^ | Not serious | 0.98 (0.64 – 1.49) | 0.9 | Not plausible | Low |
| **Stroke** | | | | | | | | | | | |
| Short-term | 7 | RCT | Not serious | Not serious | Not serious | Serious ^b^ | Not serious | 1.17 (0.68-2.02) | 0.5 | Not plausible | Moderate |
| Mid-term | 7 | RCT | Not serious | Serious ^a^ | Not serious | Serious ^b^ | Not serious | 1.25 (0.68 – 2.28) | 0.4 | Not plausible | Low |
| Long-term | 3 | RCT | Not serious | Not serious | Not serious | Serious ^b^ | Not serious | 1.09 (0.77 – 1.54) | 0.6 | Not plausible | Low |
| **Heart failure hospitalization** | | | | | | | | | | | |
| Short-term | 4 | RCT | Not serious | Not serious | Not serious | Serious ^b^ | Not serious | 0.47 (0.13-1.7) | 0.2 | Not plausible | Moderate |
| Mid-term | 5 | RCT | Not serious | Not serious | Not serious | Serious ^b^ | Not serious | 1.03 (0.64 – 1.66) | 0.9 | Not plausible | Moderate |
| Long-term | 2 | RCT | Not serious | Not serious | Not serious | Serious ^b^ | Not serious | 1.23 (0.94 – 1.63) | 0.1 | Not plausible | Low |
| **Permanent pacemaker implantation** | | | | | | | | | | | |
| Short-term | 6 | RCT | Not serious | Serious ^a^ | Not serious | Serious ^b^ | Not serious | 0.56 (0.37-0.87) | 0.008 | Not plausible | Moderate |
| Mid-term | 6 | RCT | Not serious | Not serious | Not serious | Serious ^b^ | Not serious | 0.78 (0.64 – 0.94) | 0.01 | Not plausible | Moderate |
| Long-term | 2 | RCT | Not serious | Serious ^a^ | Not serious | Serious ^b^ | Not serious | 0.86 (0.55 – 1.36) | 0.5 | Not plausible | Low |
| **Major bleeding** | | | | | | | | | | | |
| Short-term | 8 | RCT | Not serious | Not serious | Not serious | Serious ^b^ | Not serious | 0.9 (0.71-1.14) | 0.4 | Not plausible | Moderate |
| Mid-term | 4 | RCT | Not serious | Not serious | Not serious | Serious ^b^ | Not serious | 1.19 (0.71 – 2) | 0.5 | Not plausible | Moderate |
| **Valve thrombosis** | | | | | | | | | | | |
| Mid-term | 3 | RCT | Not serious | Not serious | Not serious | Serious ^b^ | Not serious | 6.25 (1.41 – 27.75) | 0.01 | Not plausible | Moderate |
| Long-term | 2 | RCT | Not serious | Not serious | Not serious | Serious ^b^ | Not serious | 5.43 (1.21 – 24.33) | 0.02 | Not plausible | Moderate |
| **Endocarditis** | | | | | | | | | | | |
| Mid-term | 3 | RCT | Not serious | Not serious | Not serious | Serious ^b^ | Not serious | 1.74 (0.71 – 4.26) | 0.2 | Not plausible | Moderate |
| Long-term | 2 | RCT | Not serious | Not serious | Not serious | Serious ^b^ | Not serious | 0.99 (0.37 – 2.69) | 0.9 | Not plausible | Low |
| **Major cardiovascular event** | | | | | | | | | | | |
| Short-term | 7 | RCT | Not serious | Not serious | Not serious | Serious ^b^ | Not serious | 0.88 (0.67-1.14) | 0.3 | Not plausible | Moderate |
| **New atrial fibrillation** | | | | | | | | | | | |
| Short-term | 3 | RCT | Not serious | Not serious | Not serious | Serious ^b^ | Not serious | 0.83 (0.55-1.25) | 0.3 | Not plausible | Moderate |
| **Acute kidney injury** | | | | | | | | | | | |
| Short-term | 6 | RCT | Not serious | Not serious | Not serious | Serious ^b^ | Not serious | 0.77 (0.54-1.09) | 0.1 | Not plausible | Moderate |
| **Effective orifice area** | | | | | | | | | | | |
| Short-term | 6 | RCT | Not serious | Serious ^a^ | Not serious | Serious ^b^ | Not serious | 0.17 (0.07-0.26) | <0.001 | Not plausible | Moderate |
| Mid-term | 5 | RCT | Not serious | Serious ^a^ | Not serious | Serious ^b^ | Not serious | 0.26 (0.11 – 0.41) | <0.001 | Not plausible | Moderate |
| Long-term | 2 | RCT | Not serious | Not serious | Not serious | Serious ^b^ | Not serious | 0.26 (0.17 – 0.32) | <0.001 | Not plausible | Moderate |
| **Mean trans-valvular pressure gradient** | | | | | | | | | | | |
| Short-term | 4 | RCT | Not serious | Serious ^a^ | Not serious | Serious ^b^ | Not serious | 3.71 (2.64-4.76) | <0.001 | Not plausible | Moderate |
| Mid-term | 6 | RCT | Not serious | Serious ^a^ | Not serious | Serious ^b^ | Not serious | 4.65 (2.95 – 6.35) | <0.001 | Not plausible | Moderate |
| Long-term | 3 | RCT | Not serious | Not serious | Not serious | Serious ^b^ | Not serious | 3.73 (3.19 – 4.27) | <0.001 | Not plausible | Moderate |
| **Moderate to severe paravalvular leak** | | | | | | | | | | | |
| Short-term | 4 | RCT | Not serious | Not serious | Not serious | Serious ^b^ | Not serious | 0.26 (0.17-0.49) | <0.001 | Not plausible | Moderate |
| Mid-term | 4 | RCT | Not serious | Serious ^a^ | Not serious | Serious ^b^ | Not serious | 0.41 (0.11 – 1.63) | 0.2 | Not plausible | Low |
| Long-term | 2 | RCT | Not serious | Not serious | Not serious | Serious ^b^ | Not serious | 0.28 (0.1 – 0.79) | <0.001 | Not plausible | Moderate |
| **Mild paeavalvular leak** | | | | | | | | | | | |
| Short-term | 3 | RCT | Not serious | Serious ^a^ | Not serious | Serious ^b^ | Not serious | 0.76 (0.51-1.14) | 0.2 | Not plausible | Low |

**Supplementary Table 5**. Quality of evidence according to GRADE framework.

1. I^2^ scores are high for the variable, suggesting significant heterogeneity between the included studies. Moreover, our subgroup analyses were unable to find the source of the heterogeneity.
2. Limited number of studies and infrequent incidence of outcomes made the results less precise.

| **Study** | **Databases** | **No. of studies** | **Short-term** | | | **Mid-term** | | | **Long-term** | | |
| --- | --- | --- | --- | --- | --- | --- | --- | --- | --- | --- | --- |
|  |  |  | In favor of BEV | In favor of SEV | Comparable | In favor of BEV | In favor of SEV | Comparable | In favor of BEV | In favor of SEV | Comparable |
| Lee et al. 2020 (1) | Medline, PubMed, Embase, and Cochrane library | 27 observational studies | PPI, and major cardiovascular events | EOA, and mean trans-valvular pressure | All-cause mortality, cardiovascular mortality, stroke, major bleeding, acute kidney injury, mild PVL, and moderate-severe PVL | - | EOA, and mean trans-valvular pressure | All-cause mortality, and stroke | NA | NA | NA |
| Hamilton et al. 2020 (2) | Medline, PubMed, and  Embase | 19 observational studies | PPI, and moderate-severe PVL | Acute kidney injury, and mean trans-valvular pressure | All-cause mortality, stroke, major cardiovascular events, and major bleeding | - | - | All-cause mortality | - | - | All-cause mortality |
| Zhang et al. 2022 (3) | PubMed, CENTRAL, and Embase | 12 observational studies and 3 RCTs | All-cause mortality, cardiovascular mortality, stroke, and moderate-severe PVL | - | PPI, major cardiovascular events, major bleeding, and acute kidney injury | All-cause mortality, and cardiovascular mortality | - | - | NA | NA | NA |
| Senguttuvan et al. 2023 (4) | PubMed, Embase, Scopus,  and Web of sciences | 6 RCTs | All-cause mortality, cardiovascular mortality, and moderate-severe PVL | - | Stroke, heart failure hospitalization, major cardiovascular events, major bleeding, and acute kidney injury | NA | NA | NA | NA | NA | NA |
| Wang et al. 2023 (5) | PubMed, Embase, and Cochrane library | 14 observational studies and 4 RCTs | PPI, mild PVL, and moderate-severe PVL | EOA, and mean trans-valvular pressure | All-cause mortality, stoke, major cardiovascular events, major bleeding, and acute kidney injury | - | - | All-cause mortality | NA | NA | NA |
| Khan et al. 2024 (6) | PubMed, Web of Science, and Cochrane library | 19 observational studies and 7 RCTs | All-cause mortality | - | Stroke, heart failure hospitalization, PPI, major bleeding, and acute kidney injury, | Heart failure hospitalization | - | All-cause mortality, stroke, | NA | NA | NA |

**Supplementary Table 6.** Main outcomes of previous meta-analyses.

BEV: Balloon-expandable valves; EOA: Effective orifice area; PPI: Permanent pacemaker implantation, PVL: Para-valvular leak; RCT: Randomized controlled trial.

1. Lee HA, Chou AH, Wu VC, Chen DY, Lee HF, Lee KT, et al. Balloon-expandable versus self-expanding transcatheter aortic valve replacement for bioprosthetic dysfunction: A systematic review and meta-analysis. PLoS One. 2020;15(6):e0233894.

2. Hamilton GW, Koshy AN, Fulcher J, Tang GHL, Bapat V, Murphy A, et al. Meta-analysis Comparing Valve-In-Valve Transcatheter Aortic Valve Implantation With Self-Expanding Versus Balloon-Expandable Valves. Am J Cardiol. 2020;125(10):1558-65.

3. Zhang XL, Wei ZH, Wang HW, Xu W, Wang Y, Xu B. Early and midterm outcomes of transcatheter aortic-valve replacement with balloon-expandable versus self-expanding valves: A meta-analysis. J Cardiol. 2022;80(3):204-10.

4. Senguttuvan NB, Bhatt H, Balakrishnan VK, Krishnamoorthy P, Goel S, Reddy PMK, et al. The safety and efficacy of balloon-expandable versus self-expanding trans-catheter aortic valve replacement in high-risk patients with severe symptomatic aortic stenosis. Front Cardiovasc Med. 2023;10:1130354.

5. Wang B, Mei Z, Ge X, Li Y, Zhou Q, Meng X, et al. Comparison of outcomes of self-expanding versus balloon-expandable valves for transcatheter aortic valve replacement: a meta-analysis of randomized and propensity-matched studies. BMC Cardiovasc Disord. 2023;23(1):382.

6. Khan QA, Farrukh AM, Belay NF, Li D, Afzal M, Nadella A, et al. Comparing outcomes of balloon-expandable vs. self-expandable valves in transcatheter aortic valve replacement: a systematic review and meta-analysis. Ann Med Surg (Lond). 2024;86(7):4060-74.
